# Exploring Determinants of Community Flood Resilience in Southeast Asia: A Systematic Review

**DOI:** 10.3390/ijerph23081013

**Published:** 2026-08-02

**Authors:** Nan Ei Moh Moh Kyi, Muhammad Naeem Rashid, Shun Lae Lae Aung, Tipsuda Pintakham, Anurak Wongta

**Affiliations:** 1 School of Health Sciences Research, Research Institute for Health Sciences, Chiang Mai University, Chiang Mai 50200, Thailand; naneimohmoh_kyi@cmu.ac.th (N.E.M.M.K.); muhammadnaeem_rashid@cmu.ac.th (M.N.R.); shunlaelae_a@cmu.ac.th (S.L.L.A.); tipsuda_pintakham@cmu.ac.th (T.P.); 2 Research Institute for Health Sciences, Chiang Mai University, Chiang Mai 50200, Thailand; 3 Environmental, Occupational Health Sciences and NCD Research Group, Research Institute for Health Sciences, Chiang Mai University, Chiang Mai 50200, Thailand

**Keywords:** floods, community flood resilience, Southeast Asia, systematic review, determinants, vulnerable populations, public health challenges

## Abstract

**Highlights:**

**Public health relevance—How does this work relate to a public health issue?**

- Flooding is Southeast Asia’s most prevalent natural disaster, disproportionately burdening vulnerable populations with injury, infectious disease, food and water insecurity, and mental health disorders.
- Community flood resilience is shaped by interacting social, environmental, physical, and psychological determinants that influence adaptive capacity and health outcomes.

**Public health significance—Why is this work of significance to public health?**

- This PRISMA 2020-registered systematic review provides the first comprehensive synthesis of mixed-methods evidence on flood resilience determinants across six Southeast Asian countries, strengthening the regional evidence base for public health planning.
- Synthesized evidence identifies psychological factors, particularly risk perception, anxiety, and self-efficacy, as cross-cutting moderators of the knowledge-to-action gap, while documenting concentrated vulnerability among older adults, low-income households, women, informal settlement residents, and rural agricultural communities.

**Public health implications—What are the key implications or messages for practitioners, policy makers and/or researchers in public health?**

- Equity-oriented disaster risk reduction strategies, which prioritize the reduction of disproportionate vulnerability among marginalized populations, should address structural determinants, including socioeconomic inequalities, infrastructure deficits, and land-use governance, alongside psychosocial barriers to strengthen community flood resilience and reduce health disparities across Southeast Asia.
- Future public health research should adopt longitudinal, gender-disaggregated, and country-comparative designs with standardized resilience measures to strengthen causal evidence and support context-sensitive policy development across the region.

**Abstract:**

Flooding is the most devastating natural disaster in Southeast Asia, disproportionately affecting vulnerable communities and posing substantial public health challenges. This PRISMA 2020 systematic review, registered with PROSPERO, aimed to synthesize the determinants influencing community flood resilience among flood-affected communities in Southeast Asia. Five electronic databases and Google Scholar were searched for empirical peer-reviewed studies published between January 2015 and January 2026. Methodological quality was independently assessed using the Kmet standard quality assessment tool, and a convergent integrated synthesis design was applied for the synthesis. Eighteen studies (nine quantitative, six qualitative, and three mixed methods) from Indonesia, Thailand, Malaysia, Cambodia, Myanmar, and Vietnam met acceptable quality thresholds. Seven determinant domains were identified: (1) sociodemographic, (2) flood exposure, (3) socioeconomic, (4) socio-cultural, (5) environmental, (6) physical, and (7) psychological factors. Synthesized evidence revealed that community flood resilience is shaped by dynamic interactions among structural, social, environmental, and psychological determinants that collectively influence resilience capacities. Psychological factors, particularly risk perception and anxiety, served as cross-cutting influences on adaptive behavior, while vulnerability remained concentrated among older adults, women, low-income households, informal settlements, and rural agricultural communities. These findings highlight the importance of integrated and equity-oriented resilience strategies that strengthen community adaptation and improve public health outcomes in the region.

## 1. Introduction

Flooding is one of the most frequent and catastrophic natural disasters across Southeast Asia, placing vulnerable populations at greater risk of adverse health and social outcomes [1]. Climate change, rapid urbanization, and environmental degradation have intensified the frequency, magnitude, and impacts of flooding, increasing its impacts on communities across the region [2]. According to the ASEAN Coordinating Centre for Humanitarian Assistance on Disaster Management, floods account for approximately 63% of all reported disaster events across ASEAN Member States [3]. A significant escalation in flood risks disrupts livelihoods, damages infrastructure, and undermines community well-being, particularly in low-lying, coastal, and riverine areas. Floods directly threaten the livelihoods of nearly 23% of the ASEAN population (approximately 146 million people) and cause substantial economic losses, particularly in Indonesia, the Philippines, Thailand, Vietnam, Malaysia, and Myanmar [4].

Beyond physical destruction, flooding poses profound public health impacts, including injury, infectious disease outbreaks, food and water insecurity, mental health disorders, and disruption of healthcare and social support services [5,6]. These burdens disproportionately affect marginalized populations with limited adaptive capacity [7]. In this review, community flood resilience is operationally defined as the collective capacity of a geographically bound community to anticipate, absorb, adapt to, recover from, and transform in response to flood events while maintaining essential community functions through shared social, organizational, and communication resources. This operationalization aligns with established community resilience frameworks by encompassing three interconnected resilience dimensions: coping, adaptive, and collaborative capacities [8,9,10]. It places greater emphasis on collective community processes than on individual or household characteristics used in some previous flood resilience assessments.

It distinguishes community resilience from individual resilience, which refers to a person’s capacity to cope with adversity, and household resilience, which reflects a family’s ability to sustain livelihoods and well-being. Community resilience emerges from interactions among individuals, households, and community systems but represents a collective property rather than the simple aggregation of individual or household capacities [11]. Identifying the determinants that shape these community-level capacities is therefore essential for reducing public health vulnerability and informing equity-focused disaster risk reduction in Southeast Asia.

The included studies conceptualized and operationalized flood resilience heterogeneously using different definitions and indicators. Some assessed resilience using household survey measures, while analyzing resilience as a community-level construct. Others examined community-level processes, such as collective action, social capital, and local adaptation through qualitative inquiry. To ensure a consistent evidence synthesis, this review adopted the above operational definition as a common conceptual framework and interpreted household-level findings as indicators of community resilience only when they were explicitly linked to broader community structures or collective adaptive processes.

In Southeast Asia, community flood resilience is mediated by interrelated social, economic, environmental, and institutional factors. Socioeconomic status, education, livelihood security, and housing quality determine preparedness and recovery capacity [2], while rapid urbanization and informal settlements amplify flood vulnerability among underprivileged groups [12]. Evidence from the Mekong basin underscores the importance of social capital and livelihood security [13,14]. However, weak governance and inadequate institutional support further constrain disaster response and adaptive capacity [15]. Equity-focused approaches addressing these structural determinants remain essential for reducing health disparities [16].

Research on flood resilience in the region remains fragmented across disciplines, methodologies, and geographical contexts. Although country-specific qualitative and mixed-methods studies have provided valuable insights [2,13,17], they offer limited understanding of regional patterns and cross-country determinants [18,19,20]. Existing literature on flood resilience in the region differs in scope, methodology, and analytical focus [2,21,22,23,24]. Lugova & Haque (2024) provided a narrative overview of community resilience in Southeast Asia but did not apply a systematic search strategy, formal quality appraisal, or structured synthesis of mixed-methods evidence [2]. Other studies in the region have primarily assessed flood resilience through empirical indices or physical, infrastructure, and health system indicators [22,24], while broader reviews have focused on flood research trends [23] or the health equity implications of flood risk outside Southeast Asia [21].

To our knowledge, no previous review has applied PRISMA 2020 guidelines with a registered protocol, dual independent quality assessment, and convergent integrated synthesis of quantitative and qualitative evidence to examine determinants of community flood resilience across Southeast Asia. This review addresses these methodological and contextual gaps by providing a comprehensive public health synthesis of resilience determinants in the region. Furthermore, factors influencing resilience in flood-affected communities remain underexplored despite evidence of substantial variation in adaptive capacities and recovery experiences across Southeast Asian settings [2,20,25,26,27].

To provide a clear theoretical foundation for this review, an integrated conceptual framework is adapted by combining the Social-Ecological Systems (SES) resilience framework and the Sendai Framework for Disaster Risk Reduction 2015–2030. The SES framework conceptualizes community flood resilience as the capacity of interconnected social, ecological, and institutional subsystems to absorb disturbances, adapt to changing conditions, self-organize, and sustain essential functions [28,29]. Within this perspective, the seven determinant domains identified in this review are organized into three interrelated components: (1) social system, (2) ecological and physical systems, and (3) governance and collective action system, consistent with place-based models of disaster resilience [30].

The social subsystem comprises sociodemographic, socioeconomic, and psychological determinants that reflect community capacities to respond to flood risk. The ecological and physical subsystem encompasses environmental and physical determinants, which define the environmental context and physical infrastructure influencing flood exposure and resilience. The collective-action subsystem consists of sociocultural determinants, including social capital, collective action, and mutual-cooperation practices, which facilitate community-level governance and coordination capacity. Flood exposure represents the external disturbance that interacts with these interconnected subsystems. The Sendai Framework complements this system’s perspective by emphasizing disaster risk understanding, risk governance, investment in resilience, and preparedness for effective response and recovery [10]. Collectively, these two frameworks provide a theoretically grounded structure for interpreting how multiple, interacting determinants shape flood resilience in Southeast Asian communities.

To address these gaps, this systematic review synthesizes evidence on the determinants of community flood resilience among flood-affected communities in Southeast Asia. Specifically, it aims to: (i) identify demographic, flood exposure, socio-economic, socio-cultural, environmental, physical, and psychological determinants of community flood resilience; (ii) examine how these determinants interact across resilience domains, assessing patterns of convergence and divergence between quantitative and qualitative evidence; and (iii) identify vulnerable population groups, experiencing differential levels of community flood resilience and related public health challenges. This evidence may inform public health intervention and disaster risk reduction policies to strengthen community flood resilience and reduce vulnerabilities in Southeast Asia.

## 2. Materials and Methods

### 2.1. Systematic Review Methodology

**Figure 1:**
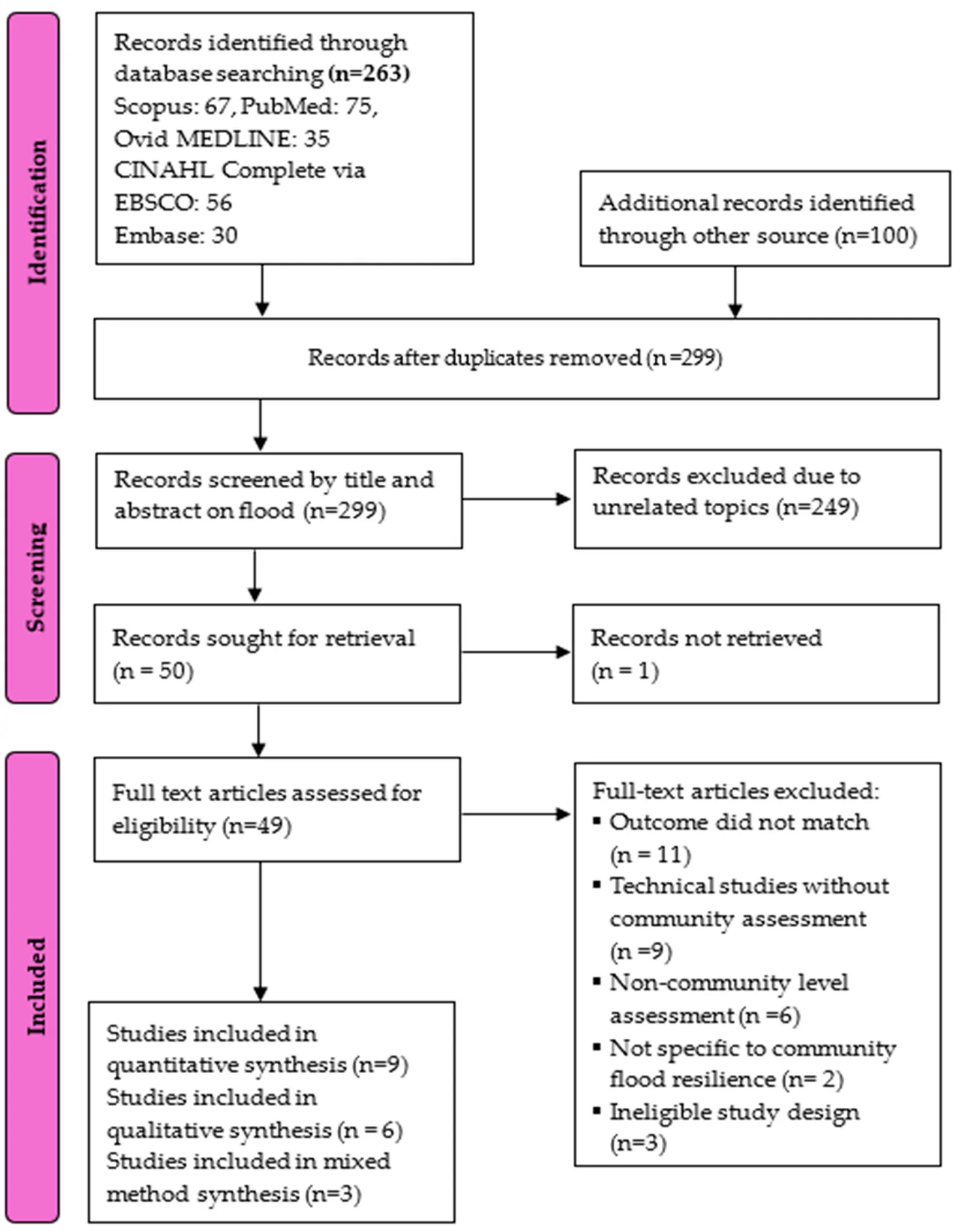
PRISMA 2020 flow diagram illustrating the study selection procedure.

This systematic review was conducted and reported in accordance with the Preferred Reporting Items for Systematic Reviews and Meta-Analyses (PRISMA) 2020 guidelines [31], and the study selection process is presented using a PRISMA 2020 flow diagram (Figure 1). The completed PRISMA 2020 checklist (Table S2) and PRISMA 2020 for abstracts checklist (Table S3) are provided in the Supplementary Materials. The protocol for this systematic review was registered in PROSPERO (Reference: CRD420261286656).

### 2.2. Eligibility Criteria

This systematic review included peer-reviewed primary studies conducted among flood-affected communities in rural or urban settings across Southeast Asian countries. Studies were eligible for inclusion if they analyzed key determinants influencing community flood resilience and assessed at least one associated factor, including demographic, socioeconomic, sociocultural, environmental, physical, and psychological factors. Studies employing cross-sectional or comparative, cohort or longitudinal, qualitative, or quantitative research designs were included. Only full-text articles published in English-language, peer-reviewed journals published between January 2015 and January 2026 (up to the date of the final literature search) were considered. Translation services were not employed due to resource constraints, and this eligibility criterion was predefined in the review protocol. We acknowledge that this language restriction may have excluded relevant studies published in Southeast Asian languages and introduced language bias.

Studies were excluded if they: (i) were conducted outside Southeast Asia, in non-flood-affected communities, or among general populations without a community-level analysis; (ii) focused only on flood impacts, damage, or health outcomes without examining community resilience or its determinants; (iii) examined exposures unrelated to flooding or community resilience; (iv) focused exclusively on technical, ecological, hydrological, or environmental processes without community involvement; and (v) were not primary empirical research (e.g., reviews, editorials, or policy briefs). Grey literature, including theses, dissertations, technical or organizational reports, and conference abstracts, was also excluded to ensure that the review focused exclusively on peer-reviewed scientific articles.

### 2.3. Sources of Information and Search Strategy

A systematic search was conducted in Scopus, PubMed, MEDLINE via Ovid, Embase, and CINAHL Complete via EBSCO. Google Scholar was searched as a supplementary source to identify grey literature and additional relevant studies not retrieved in traditional databases. The first 100 records, sorted by relevance and publication years, were screened using Publish or Perish (PoP) software (Version 8.19.5300.9483) to maintain transparency in evidence identification [32,33].

The keywords used to search for articles were communit* OR community level* OR community-based OR household* OR village* OR settlement* OR local communit* OR flood-affected communit* OR neighborhood* OR flood affected household* AND flood* OR inundation OR flood disaster* OR flood hazard* OR flood exposure* OR flood risk* OR river overflow* OR “flash flood*” OR “monsoon flood*” OR “urban flood*” OR “coastal flood*” OR extreme rainfall AND factor* OR determinant* OR predictor* OR driver* OR influence* OR socio-demograph* OR socio-econom* OR socio-cultur* OR environment* OR physical* OR psychological* OR “mental health*” OR “wellbeing*” OR “social capital*” OR livelihood* AND resilien* OR “community resilience” OR “community adaptation” OR “flood resilience” OR “coping capacity” OR “adaptive capacity” OR “recovery capacity” OR “flood preparedness*” OR “response capacity” AND “Southeast Asia” OR “South-East Asia” OR “Southeastern Asia” OR “Mekong Region*” OR ASEAN OR Thailand OR Myanmar OR Vietnam OR Cambodia OR Laos OR Malaysia OR Indonesia OR Philippines OR Singapore OR Brunei OR “Timor-Leste”. Searches were limited by publication year.

### 2.4. Study Selection Process

The PRISMA flowchart follows the four main stages of the systematic review process: study identification, screening, eligibility assessment, and inclusion. All retrieved records were exported to EndNote 21 for reference management and screening. Duplicate records were identified and removed prior to the screening process. Study selection was conducted independently by two authors, with discrepancies resolved through discussion or, when necessary, consultation with a third author.

During the screening phase, two authors independently applied a two-stage screening process. In the first stage, titles and abstracts were screened to assess relevance to the review question and to exclude studies that were clearly unrelated to flood-related topics or conducted outside the Southeast Asian region. Eligible studies were required to involve human populations affected by flooding within a Southeast Asian context. Articles addressing floods either as a sole hazard or in combination with other climate-related hazards (e.g., droughts or storms) were included if they reported community-level impacts or examined concepts related to flood resilience. Review articles, editorials, and grey literature were also excluded at this stage in accordance with the predefined eligibility criteria. In the second stage, full-text articles that met the initial screening were assessed in detail for eligibility assessment based on predefined inclusion and exclusion criteria.

### 2.5. Assessment of Methodological Quality

The risk of bias or methodological quality of included studies was independently assessed by two authors using standard quality assessment criteria [34]. Each item was scored as “yes” (2 points), “partial” (1 point), or “no” (0 points). For quantitative studies, items that were not applicable to the study design were recorded as “not applicable (N/A)” and excluded from the denominator when calculating the overall quality score. The total quality score was calculated by summing the scores of all applicable items, dividing by the maximum possible score, and multiplying by 100 to obtain a percentage score (obtained score/total possible score × 100).

Any disagreements between reviewers were resolved through discussion and re-evaluation of the original studies. If consensus could not be reached, a third reviewer was consulted for final resolution. For mixed-methods studies, quantitative and qualitative components were appraised separately using the corresponding Kmet et al. assessment criteria. For example, Yang et al. (2024) employed a longitudinal time-series design for its quantitative component, which was evaluated using the quantitative Kmet checklist as a longitudinal observational study, while its qualitative focus-group discussion and key informant interview components were assessed independently using the qualitative checklist [34,35]. The time-series design was retained as originally reported and was not reclassified for the purposes of quality appraisal. The overall methodological quality was determined based on a predefined criterion requiring both components to meet the minimum quality threshold to ensure adequate quality of each methodological component.

Studies with a quality score of ≥55% were included in the systematic review. Kmet et al. (2004) proposed 55% as a relatively liberal inclusion threshold, compared with a more conservative 75% cut-off. We adopted the 55% threshold to retain relevant observational and mixed-methods evidence from disaster and public health research in Southeast Asia while excluding studies with substantial methodological limitations. Study quality was subsequently categorized as high (≥75%), moderate (55–74%), and low (<55%) [34].

### 2.6. Data Extraction

Data from eligible studies were systematically extracted by one author and verified by another to ensure accuracy. A structured data extraction matrix was developed in Microsoft Excel to capture relevant information from all included studies. Extracted information included author(s), year of publication, country/study setting, study design, study population and sample size, sampling technique, data collection, data analysis, key findings, and quality appraisal. All data was organized in an Excel matrix to maintain transparency and reproducibility.

### 2.7. Data Synthesis

There are several approaches to data synthesis in systematic reviews, particularly when integrating qualitative and quantitative evidence. This review applied a convergent integrated synthesis design, in which quantitative and qualitative evidence were synthesized independently using design-appropriate methods [36]. Quantitative findings were synthesized narratively by grouping study results according to the predefined determinant domains. A meta-analysis was not undertaken due to substantial heterogeneity in study designs, resilience outcome measures, populations, and operational definitions of resilience across the included studies. Qualitative findings were synthesized by comparing and categorizing findings with similar meanings into the corresponding determinant domains. Rather than conducting a formal coding process, two authors independently grouped similar concepts and reported determinants to identify common patterns across studies, with disagreements resolved through discussion.

The synthesized quantitative and qualitative findings were then integrated using a comparative convergence approach [36], in which the findings from both sources were compared within each determinant domain to identify areas of convergence (consistent findings), complementarity (findings providing additional contextual explanations), or divergence (apparently conflicting findings). When discrepancies were identified, they were interpreted by considering differences in study characteristics, population, and context rather than prioritizing one type of evidence over another. An overall interpretation was subsequently developed across all determinant domains [37]. The general characteristics of the included studies are presented in (Table 1). A concise summary of the synthesized evidence is presented in the manuscript (Table 2), while the detailed comparative convergence synthesis table is provided in the Supplementary Materials (Supplementary Table S1).

## 3. Results

### 3.1. Study Selection

The database search identified 263 records (Scopus: 67; PubMed: 75; MEDLINE via Ovid: 35; CINAHL via EBSCO: 56; Embase: 30). An additional 100 records were identified through supplementary Google Scholar searching, yielding a total of 363 records. After removing 64 duplicates, 299 records remained for title and abstract screening. Of these, 249 were excluded for failing to meet the eligibility criteria. The remaining 50 records were sought for full-text retrieval; however, one record could not be accessed. A total of 49 full-text articles were assessed for eligibility, of which 31 were subsequently excluded with specific reasons: outcome not focused on community flood resilience determinants (*n* = 11); technical studies without community assessment (*n* = 9), no community-level analysis (*n* = 6); not specific to community flood resilience (*n* = 2); and experimental or non-empirical design (*n* = 3). Ultimately, 18 primary studies fulfilled all eligibility criteria and were selected for qualitative narrative synthesis. The complete study screening and selection flow is illustrated in the PRISMA 2020 flow diagram (Figure 1).

### 3.2. Study Characteristics

Eighteen studies published between January 2015 and January 2026 were conducted in six countries: Indonesia (*n* = 9), Thailand (*n* = 3), Malaysia (*n* = 2), Cambodia (*n* = 2), Myanmar (*n* = 1), and Vietnam (*n* = 1). No eligible studies were identified from Laos, the Philippines, Singapore, Brunei, or Timor-Leste. Study settings comprised coastal (*n* = 6), urban (*n* = 4), rural/peri urban (*n* = 5), and riverine (*n* = 3) communities.

By study design, nine studies were quantitative cross-sectional, with one employing structural equation modeling (SEM), six were qualitative (using phenomenology, in-depth interviews, focus groups, interpretive case studies, and narrative analysis), and three used sequential explanatory mixed methods. Fifteen studies examined community members or households in flood-prone areas; two focused on older people (*n* = 1) and slum households (*n* = 1). Sample sizes ranged from 7 to 1398 participants. Data collection relied predominantly on questionnaires and semi-structured interviews, supplemented by focus group discussions, field observations, and document or social media analysis. Quantitative studies employed descriptive statistics, correlation, regression, factor analysis, and structural equation modeling. Qualitative studies used thematic, phenomenological, narrative, and content analysis (Table 1).

**Table 1:** General characteristics of the studies included in the systematic review (*n* = 18).

No.	First Author (Year)	Country/Setting	Study Design	Study Population (n) and Sampling	Data Collection and Data Analysis
1	Kumaresen, M, et al. (2025) [38]	Malaysia (Urban, Greater Kuala Lumpur)	Quantitative Cross-sectional	*n* = 212; Purposive	▪ Questionnaire ▪ Descriptive statistics; composite mean index; Spearman correlation
2	Lwin, K.K., et al. (2020) [39]	Myanmar (Rural, Ayeyarwady Delta)	Quantitative Cross-sectional	*n* = 215 households (high/low flood risk areas); Random	▪ Household questionnaire ▪ Descriptive stats; *t*-test; Chi-square; Pearson correlation
3	Isa, M., et al. (2018) [40]	Indonesia (Coastal, North Coast of Central Java)	Quantitative Cross-sectional	*n* = 390; Multistage	▪ Questionnaire (face-to-face), stakeholder in-depth interviews ▪ Multinomial logistic regression
4	Thapa, S., et al. (2025) [41]	Thailand (Urban, Songkhla Old Town)	Quantitative Cross-sectional	*n* = 450 households; Systematic random	▪ Questionnaire (face-to-face) ▪ Multiple regression; VIF/multicollinearity check (SPSS v29)
5	Nugraheni, I. L., et al. (2022) [42]	Indonesia (Peri-urban/rural, Lampung)	Quantitative Cross-sectional	*n* = 1398 household heads; Random	▪ Questionnaire (interviews); ▪ Structural Equation Modelling (SEM); Confirmatory Factor Analysis (CFA)
6	Mruksirisuk, P., et al. (2023) [43]	Thailand (Urban/peri-urban, Pathum Thani)	Quantitative Cross-sectional	*n* = 494 households; Random	▪ Expert-validated questionnaire; flood/climate/GIS data ▪ SEM; CFA; FVI scoring
7	Saputra, H., et al. (2025) [44]	Indonesia (Coastal rural, Madura Island)	Quantitative Cross-sectional	*n* = 399 older persons (≥60 yrs); stratified random	▪ Questionnaire; face-to-face interviews ▪ Descriptive statistics; EFA; PCA; Spearman correlation
8	Ningtias, Y.R., et al. (2025) [45]	Indonesia (Riverine urban, Surakarta City)	Quantitative Cross-sectional	*n* = 87; Stratified random	▪ Questionnaire ▪ Multiple linear regression (OLS)
9	Ludin, S.M., et al. (2019) [46]	Malaysia (Rural, Kelantan)	Quantitative Cross-sectional	*n* = 386 evacuees; Systematic household	▪ Questionnaire (assisted administration) ▪ Descriptive stats; one-way ANOVA; Pearson correlation
10	Aksa, F. I., et al. (2022) [47]	Indonesia (Coastal, Langsa City, Aceh)	Qualitative In-depth interview	*n* = 50 community members (village heads, youth leaders, residents); Purposive	▪ In-depth interviews; field observation (participatory) ▪ Descriptive stats; qualitative interpretation
11	Priyanti, R. P., et al. (2019) [48]	Indonesia (Riverine, East Java)	Qualitative Phenomenology	*n* = 7; Purposive	▪ Semi-structured interviews; observation; field notes ▪ Colaizzi phenomenological analysis
12	Marfai, M. A., et al. (2015) [49]	Indonesia (Urban coastal, Jakarta)	Qualitative In-depth interview	*n* = 128; Purposive (participatory approach)	▪ In-depth interviews; field observations ▪ Qualitative descriptive (inductive)
13	Dwinantoaji, H., et al. (2020) [50]	Indonesia (Urban coastal, Semarang)	Qualitative Focus Group Discussion	*n* = 22; Purposive	▪ Focus group interview ▪ Qualitative content analysis (NVivo 10)
14	Leong, C. M. L., et al. (2015) [51]	Thailand (2011 flood-affected multi-sites)	Qualitative Case study (interpretive)	*n* = 56; Purposive	▪ Semi-structured interviews; FGDs; archival social media/document analysis ▪ Iterative interpretive analysis
15	Tran, T.A., et al. (2022) [52]	Vietnam (Rural agrarian, Mekong Delta)	Qualitative Narrative analysis	9 FGDs; *n* = 51 interviews; Purposive/ snowball	▪ FGDs; in-depth interviews ▪ Thematic & narrative analysis (NVivo)
16	Yang, Y., et al. (2024) [53]	Cambodia (Rural, Battambang)	Mixed-methods Longitudinal time-series (T0-T1)	*n* = 300 households; FGD (*n* = 19); KII (*n* = 9)	▪ Questionnaire ▪ FRMC scoring; time-series comparative analysis (T0 vs. T1); thematic analysis
17	Yang, J., & Andriesse, E. (2021) [17]	Cambodia (Riverine slum urban, Phnom Penh)	Mixed methods Sequential explanatory	*n* = 119 households; *n* = 25 interviews; Random/snowball	▪ Household survey; semi-structured interviews ▪ Qualitative coding and quantitative pathway analysis
18	Markolinda et al. (2025) [54]	Indonesia (Coastal rural, Mentawai Islands)	Mixed methods Sequential explanatory	*n* = 106; 9 KII	▪ Semi-structured interviews ▪ Descriptive stats; thematic analysis; triangulation

Abbreviations: VIF = Variance Inflation Factor; SEM = Structural Equal Modelling; CFA = Confirmatory Factor Analysis; FVI = Flood Vulnerability Index; EFA = Exploratory Factor Analysis; PCA = Principal Component Analysis; OSL = Ordinary Least Squares; FGD = Focus Group Discussion; KII = Key Informant Interview.

### 3.3. Methodological Quality Assessment

All included studies exceeded the predefined quality threshold of 55% and were retained for evidence synthesis. Among the quantitative components of twelve studies, including nine quantitative studies and quantitative strands of three mixed-methods studies, the quality score ranged from 63.64% to 100.0% (14-item scale, max 22). Among the qualitative components of nine studies, comprising six qualitative studies and qualitative strands of three mixed-methods studies, the scores ranged from 65.0% to 90.0% (10-item scale, max 20). Detailed item-level quality assessment results were provided in Appendix A Table A1 and Table A2.

### 3.4. Synthesized Determinants of Community Flood Resilience by Domains

The analysis identified determinants of community flood resilience across seven primary domains, including socio-demographic, exposure to flooding, socio-economic, socio-cultural, environmental, physical, and psychological factors. Across the included quantitative studies, a range of statistical approaches was used to evaluate determinants of community flood resilience. These included correlation analyses (*r* = 0.53–0.74), standardized regression models (*β* = 0.13–0.66), chi-square tests (χ^2^ = 9.84–31.62), and composite index or indicator scores (approximately 0.17–0.90), with most associations reaching statistical significance ranging from *p* < 0.05 to *p* < 0.001. Table 2 presents a narrative summary of key determinants by domain, while Supplementary Table S1 provides the corresponding domain-specific effect sizes, statistical values, and a comprehensive convergence synthesis of quantitative and qualitative evidence.

**Table 2:** Synthesis of determinants influencing community flood resilience by domain (*n* = 18).

Determinants by Domain	Supporting Studies	Synthesized Interpretation
Sociodemographic ▪ Age ▪ Household size ▪ Proportion of household dependents ▪ Education ▪ Health status	7 studies Quanti: [39,40,41,43,44] Quali: [48] Mixed-methods: [17]	▪ Quantitative and qualitative evidence identify older age as a vulnerability factor, though qualitative insight remains limited regarding age-specific adaptation pathways and resilience mechanisms. ▪ Larger households and high concentration of dependents increase flood vulnerability through compounded caregiving and resource demands during sudden-onset evacuations. ▪ While quantitative findings showed no significant association between education and resilience capacity, qualitative evidence indicated that low education posed path-dependent coping, reduced risk awareness, and limited capacity to pursue transformative adaptation strategies. ▪ Complementary evidence: Quantitative component loadings demonstrate the structural necessity of health assets, while qualitative data highlights how disease burdens directly deplete community savings.
Flood exposure ▪ Flood frequency, depth, duration ▪ Riverine/coastal proximity ▪ Prior flood experience	8 studies Quanti: [39,40,41,43] Quali: [47,48,49,52]	▪ Quantitative and qualitative evidence converges on the structural severity of flood exposure: statistical damage coefficients match qualitative narratives. ▪ Evidence diverges on behavioral and cognitive pathways: quantitative findings associate exposure with elevated risk perception while qualitative findings indicate that preparedness depends on social memory, place attachment, and community capacity rather than exposure frequency alone. ▪ Complementary: transformative adaptation emerges where cumulative experience intersects with social cognition, not from exposure itself.
Socioeconomic ▪ Income level ▪ Debt ▪ Financial capital ▪ Livelihood diversification/dependence ▪ Employment status ▪ Housing condition	6 studies Quanti: [39,43] Quali: [47,52] Mixed-methods: [17,35]	▪ Both bodies of evidence converge on low income, debt, weak financial capital, and limited livelihood diversification as constraints on adaptive capacity and amplifiers of flood sensitivity. ▪ Quantitative indices map exposure and sensitivity thresholds; qualitative findings explain how daily-wage instability and debt generate cumulative, path-dependent vulnerability, which prevents housing investment and channels households toward small-scale coping. ▪ Complementary: quantitative metrics capture structural deficits, while qualitative accounts demonstrate communal resources as compensatory, but insufficient adaptation.
Sociocultural ▪ Social capital ▪ Community cohesion Collective action ▪ Community participation ▪ Mutual cooperation (gotong royong) ▪ Social support ▪ Indigenous knowledge ▪ Social media ▪ Cultural practices	13 studies Quanti: [38,39,41,42,45,46] Quali: [48,49,50,51,52] Mixed-methods: [35,53]	▪ Both evidence consistently identify social capital, cohesion and support as primary resilience enablers across Southeast Asian settings. ▪ Quantitative metrics quantify predictive strength, while qualitative evidence reveals activation mechanisms (mutual cooperation, cultural practices, ICT-driven mobilization) that transform latent social capital into protective action during flood crises. ▪ Quantitative frameworks measure social capital as generic network capacity; qualitative evidence specifies how indigenous knowledge and cultural practices enable transformative adaptation distinct from formal institutional responses.
Environmental ▪ Land-use ▪ Infrastructure quality ▪ Natural capital	6 studies Quanti: [42,43] Quali: [47,49,52] Mixed-method: [35]	▪ Land-use change, degraded drainage infrastructure, and ecosystem decline constitute structural drivers of flood vulnerability in both evidence streams. ▪ Quantitative factor loadings quantify systematic vulnerability, while qualitative evidence further enriches how environmental stressors convert predictable seasonal floods into frequent, unmanageable tidal inundations that overwhelm traditional coping thresholds. ▪ Quantitative natural capital scores stagnate, while qualitative evidence identifies ecosystem-based adaptation as a pathway translating social memory into resilience-building responses.
Physical ▪ Building quality ▪ Utility disruptions ▪ Drainage adequacy ▪ Physical capital	5 studies Quanti: [38,43] Quali: [48,49] Mixed-method: [35]	▪ Convergence on infrastructure deficits: Building quality, utility disruptions, and drainage inadequacy heighten flood sensitivity and depress adaptation. ▪ Quantitative indices measure deficit severity, while qualitative evidence reveals how communities compensate through human-centered early warning systems and collective mobilization. ▪ Divergent: Quantitative scores indicate persistent infrastructural deficiency; qualitative findings demonstrate that community-led adaptations remain insufficient for sustained resilience.
Psychological ▪ Preparedness knowledge ▪ Flood risk perception ▪ Perceived consequences ▪ Anxiety ▪ Emotional confidence ▪ Fear ▪ Self-efficacy ▪ Trust	6 studies Quanti: [39,41,44] Quali: [47,48,51]	▪ Convergent evidence confirms that knowledge, perception, and trust promote preparedness, whereas anxiety and fear reduce it. ▪ Quantitative models identify risk perception as a direct predictor, while qualitative evidence reveals that awareness alone is insufficient when place attachment and economic dependence restrict choices. ▪ Complementary: Coefficients identify trust and knowledge as drivers, while qualitative evidence explains how trust erodes through negative experiences, but rebuilds through ICT-mediated self-efficacy and collective action.

#### 3.4.1. Socio-Demographic Determinants

Five socio-demographic determinants were identified across seven studies: age, household size, proportion of household dependents, education, and health status. Older age consistently emerged as a vulnerability factor, being associated with physical limitations during flood events (*r* = 0.68, *p* < 0.001). Qualitative findings further identified older adults as a group requiring targeted support, although evidence on age-related adaptation and resilience pathways remains limited [44]. Household structure also influenced vulnerability; larger household size and a high proportion of dependents increased flood vulnerability by intensifying caregiving, evacuation, and resource demands during flood events [40,41,43,48].

Although education was not significantly associated with resilience capacities in quantitative analyses, qualitative findings revealed that education strengthened adaptive capacity through knowledge and training, while limited education reinforced short-term coping and constrained long-term adaptation [17]. Health status was also identified as an important resilience determinant, with health condition and physical mobility contributing to resilience at loadings of 0.168 and 0.171 [44]. Qualitative evidence further indicated that injury, disease burden, disability, and disrupted coping practices amplified vulnerability and placed additional pressure on household resources [17,39,41].

#### 3.4.2. Determinants of Flood Exposure

Flood exposure characteristics, including flood frequency, depth, duration, riverine and coastal proximity, and prior flood experience, were identified as key determinants shaping community flood resilience in eight studies. Physical flood exposure significantly predicted resilience (*β* = 0.354; OR = 1.425; *p* = 0.001), with damage and losses showing the greatest influence (Resilience Indicator [RI] = 0.94 vs. 0.93), followed by casualties (RI = 0.69) [40]. Prolonged flood depth and duration also heightened structural damage (*r* = 0.741, *p* < 0.01) [43] and destroyed local road networks, halting physical access and disrupting emergency distributions [49]. Proximity to water bodies was a significant geographical predictor (*β* = 0.312, *p* < 0.001) [40,41]. Communities with high exposure reported higher risk perception (Weighted Average Index [WAI] = 0.90) but lower asset capital (WAI ≤ 0.68), although social resilience and adaptive capacity remained relatively strong (WAI ≥ 0.68) [39].

Quantitative evidence associated flood exposure with elevated resilience, vulnerability, damage and risk perception [39,40,41,43]. Qualitative findings revealed that flood preparedness depended on social memory, cognition, and place attachment rather than exposure alone [47,48,49,52]. Repeated flood events built social memories that connected past experience to present adaptation, occasionally supporting transformative adaptation [48,52]. Despite high-risk awareness, formal preparedness behaviors remained low due to limited community capacity [47,48]. These findings converge on the structural effects of exposure in which statistical damage coefficients matched qualitative narratives of isolated urban areas during prolonged inundation but diverge in behavioral and cognitive pathways linking experience to preparedness.

#### 3.4.3. Socio-Economic Determinants

Household income, debt, financial capital, livelihood conditions, and housing were identified as barriers to flood resilience across six studies. Low income and household debt increased flood exposure (index = 0.531) and sensitivity (index = 0.633), while adaptive capacity was constrained by household income (index = 0.503) [43]. In contrast, higher income was associated with greater social resilience (Chi-square = 31.62, *p* < 0.001) [39]. Qualitative evidence further identified limited financial capital as a major barrier to resilience among poor households [35].

Livelihood insecurity compounded socio-economic vulnerability. Informal employment was associated with low adaptive capacity (*p* < 0.05) [43], while qualitative narratives linked informal livelihoods to unstable income, debt accumulation, absence of safety nets, and post-flood income loss [17]. Economic dependency, limited livelihood diversification, and poor housing conditions restricted adaptation options and reinforced reliance on small-scale, self-help coping strategies [17,47,52]. In addition, poor housing conditions intensified vulnerability, as low-income households had limited capacity for structural adaptations [17]. Daily-wage instability prevented investment in permanent housing improvements [35,43]. The combined evidence indicated that financial constraints and limited livelihood diversification weakened adaptive capacity and increased flood sensitivity to flood impacts, with qualitative findings additionally explaining how financial capital produced cumulative, path-dependent vulnerability over time.

#### 3.4.4. Sociocultural Determinants

Sociocultural determinants, encompassing social capital, community cohesion, participation, collective action, mutual cooperation (gotong royong), social support, indigenous knowledge, social media, and cultural practices, were consistently reported as flood resilience factors across 13 studies. Quantitative results identified social capital as a strong predictor of flood preparedness (*β* = 0.659, *p* < 0.01) [45] and the highest adaptive capacity component (mean index = 7.86) [38]. Community cohesion was linked to adaptive capacity in high- and low-flood-prone areas (Weighted Average Index (WAI) ≥ 0.68) [39], while collective action, trust, and social networks strengthened social cohesion (*p* < 0.01) [46]. Additionally, community participation was correlated with social cohesion (*r* = 0.529, *p* < 0.01) [46], and active engagement significantly improved flood resilience (*p* < 0.01) [39]. Social support further predicted household flood preparedness (*β* = 0.134, *p* < 0.01) [41].

Qualitatively findings complemented these results by verifying social capital, leadership, stakeholder coordination, and family and community attachment as mechanisms that strengthened preparedness, reduced vulnerability, and enhanced resilience [48,49]. Mutual cooperation (gotong royong), organizational support, and community participation also enhanced collective response capacities [47,50], while ICT and social media facilitated community empowerment through information access and collective action [51]. Indigenous knowledge and cultural practices further supported adaptation and transformative resilience [52,53]. Both evidence streams converged on social capital, community cohesion, and social support as major factors of flood resilience in Southeast Asian settings.

#### 3.4.5. Environmental Determinants

Environmental influences were represented across three interrelated factors, comprising land-use patterns, infrastructure quality, and natural capital. Quantitative findings demonstrated that land-use conversion, drainage systems, and ecosystem condition were primary structural drivers of systematic flood vulnerability. The confirmatory factor analysis (CFA) showed strong contributions of land-use patterns (factor loading = 0.704) and substandard drainage systems (factor loading = 0.789) [43], whereas the Conversion of Land Use and its Effects at Small regional extent (CLUE-S) model further confirmed the impact of land-use change on flood resilience (Kappa coefficient [*κ*] = 0.581) [42]. Moreover, natural capital, measured as ecosystem condition, remained a major driver of flood vulnerability, with no improvement recorded over the study period (Flood resilience measurement for communities [FRMC] score = 33/100) [35].

Complementing these findings, four qualitative and mixed-method studies illustrated the process that intensified flooding and reduced traditional community coping capacity [47,49]. Poor drainage quality, inadequate infrastructure and maintenance contributed to small-scale, uncoordinated adaptation and amplified flood exposure [49], while ecosystem-based adaptation further supported resilience-building by translating social memory into adaptive responses [52].

#### 3.4.6. Physical Determinants

Building quality, utility disruptions, drainage adequacy, and physical capital were identified as flood resilience determinants in five studies. Poor building quality and severe utility disruptions were highlighted as primary drivers of flood sensitivity, with sensitivity indices of 0.611 and 0.747, while warning and assistance measures exhibited the lowest adaptive capacity performance (converted score = 0.257) [43]. In addition, adequate drainage was rated as the lowest-scoring preparedness item (Mean = 3.62), reflecting a persistent infrastructural deficiency [38]. Physical capital deficits, including limited access to safe water and energy supply, recorded at 17–33% below standard, further reduced flood resilience, compounded by financial stagnation and low natural capital [35].

With evidence from three qualitative and mixed-method studies, poor drainage, weak infrastructure quality, and inadequate maintenance amplified flood exposure and resulted in fragmented, localized adaptation responses [49]. Communities frequently relied on human-centered early warning systems and collective mobilization to compensate for physical infrastructure limitations [48]. Both evidence streams confirmed that building quality, drainage, utilities, and physical infrastructure influenced flood sensitivity and adaptive capacity, while qualitative findings further elaborated that community-led responses alone were insufficient to sustain long-term resilience.

#### 3.4.7. Psychological Determinants

Six studies examined psychological determinants, including preparedness knowledge, flood risk perception, perceived consequences, anxiety, emotional confidence, fear, self-efficacy, and trust in emergency systems [39,41,44,47,48,51]. Quantitative evidence showed preparedness knowledge as the strongest predictor of proactive adaptation (*β* = 0.539), followed by perceived risk (*β* = 0.202) and perceived consequences (*β* = 0.193), at *p* < 0.01 [41]. Conversely, anxiety reduced preparedness (*β* = −0.124, *p* = 0.010) [41], while low confidence in emergency rescue systems (*β* = −0.174) and fear of self-evacuation (*β* = −0.245) diminished resilience among elderly populations, at *p* < 0.01 [44]. Higher trust in officials and emergency services (χ^2^ = 9.84, *p* < 0.01), combined with flood risk awareness and actionable flood protection knowledge, further promoted social resilience [39].

Qualitative evidence revealed that risk awareness alone was insufficient to drive proactive adaptation, as place attachment and socio-economic dependency restricted relocation decisions [47]. Negative flood experiences and weak infrastructure reinforced perceptions of powerlessness and community passivity [48], whereas ICT and social media enhanced self-efficacy and supported collective action [51]. Synthesized evidence confirmed that knowledge, risk perception, trust, and self-efficacy strengthened preparedness and resilience, whereas anxiety, fear, and social dependency constrained adaptive responses. ICT and social networks further supported collective resilience.

#### 3.4.8. Vulnerable Populations and Public Health Challenges

The study identified five population groups experiencing disproportionate flood vulnerability (Table 3). Older adults were vulnerable due to physical limitations, mobility dependence, chronic conditions, loneliness, and low education. During floods, they often experienced evacuation difficulties, restricted healthcare access, and disrupted care continuity, contributing to poor physical and psychological health. Their resilience relied on accessible healthcare, institutional support, and family networks [35,39,43,44,46]. Among older people on Madura Island, age was strongly correlated with physical limitations (*r* = 0.68, *p* < 0.001), reflecting the mobility constraints associated with older age [44]. Additionally, low-income households exhibited a multifaceted vulnerability, driven by financial insecurity, low education, and informal employment. These constraints forced families into high-debt cycles and poor-quality housing in flood-prone areas. Such conditions triggered public health challenges, including reduced access to medical care, inadequate preparedness supplies, and compromised sanitation. Ultimately, these factors severely undermined adaptation and recovery capacity after flood disasters [17,43,44].

Similarly, women faced distinct vulnerabilities during flood events, arising from unequal resource access and heavy caregiving responsibilities, which often led to social isolation and limited financial independence. These factors created a disproportionate care burden that hindered effective evacuation and recovery while contributing to increased psychological stress [35,39,44]. However, evidence specifically regarding female-headed households was limited. Although female household headship has been identified as a potential vulnerability indicator, Thapa et al. (2025) found that it was not significantly associated with flood preparedness, suggesting that gender-related vulnerability may be more closely linked to caregiving responsibilities, unequal resource access, and exclusion from decision-making than to household headship alone [35,41]. Informal settlements and slum households also encountered insecure land tenure, inadequate infrastructure and basic services, and exclusion from formal emergency responses. Limited access to clean water, sanitation, shelter, and medical services compromised their health, compounded by high population density and financial deficits. However, the communities navigated these constraints through gotong royong (mutual cooperation) and community-based adaptation [17,47,48,49].

Rural agricultural communities experienced livelihood disruption food and water insecurity from repeated flooding. Changing hydrological patterns, lack of protective mitigation structures, and restricted access to resources further increased their vulnerability. Furthermore, these communities frequently suffered physical and psychological trauma and loss of cultural identity from landscape transformations, which has been worsened by poor access to healthcare and essential services [39,52].

## 4. Discussion

### 4.1. Summary of Key Findings

This study synthesized the evidence from 18 peer-reviewed studies across six Southeast Asian countries to examine the determinants of community flood resilience in flood-affected areas over the past decade. The convergent evidence indicates that flood resilience is shaped by dynamic interactions among demographic, socioeconomic, sociocultural, environmental, and community-level adaptive processes, with psychological factors acting as a cross-cutting moderator of the knowledge-behavior gap. These interactions produced unequal resilience outcomes, disproportionately burdening disadvantaged populations. Strengthening integrated interventions that address psychological barriers alongside structural determinants is critical for addressing disaster risk reduction and public health issues in the region.

### 4.2. Determinants Influencing Community Flood Resilience in Flood-Affected Areas of Southeast Asia

The synthesized evidence supports a multidimensional understanding of community flood resilience in which the seven determinant domains operate as interconnected components of a social-ecological system (SES) rather than independent predictors of resilience. Consistent with the SES resilience framework, community resilience emerges through interactions among three interrelated subsystems: the social subsystem (sociodemographic, socioeconomic, and psychological determinants), the governance and collective-action subsystem (sociocultural determinants), and the ecological and physical subsystem (environmental and physical determinants), while flood exposure represents the external disturbance to which these subsystems respond. Taken together, these domains influence communities’ capacities to prepare for, cope with, adapt to, and recover from flooding through dynamic interactions across individual, community, and environmental levels.

Within the social subsystem of the SES framework, sociodemographic characteristics play a crucial role in determining community vulnerability and resilience to flood disasters. Although several determinants identified in the included studies were measured at the individual or household level (e.g., age, education, and household composition), all included studies assessed resilience at the community level. Therefore, these determinants were interpreted as contributors to community resilience rather than as measures of individual or household resilience, consistent with the review eligibility criteria and the Social-Ecological Systems framework. This review discovered that the elderly were at risk of flood vulnerability due to their limited mobility, dependency, and chronic health conditions, which is consistent with evidence on contemporary social vulnerability literature [54,55]. However, an important gap remains in understanding how older adults transition from vulnerability to active resilience.

The protective role of education contributed to improving disaster information access, risk understanding, and facilitating engagement in preparedness and recovery activities. The finding is supported by prior evidence suggesting that education reduces disaster vulnerability by strengthening community response capacity [56]. Qualitative findings further revealed that education may support long-term adaptation, whereas limited education may reinforce reliance on short-term coping [17]. Moreover, household structure affected disaster resilience, as large households and high dependency ratios intensified caregiving and evacuation burden during flooding [41,44]. Particularly, in Indonesia and Thailand, where multigenerational contexts are widespread, older and younger dependents rely on working-age members. Mobility limitation and chronic health conditions further compound these constraints through reduced evacuation and response capacity [57].

However, across many determinants, quantitative studies frequently reported no statistically significant associations, whereas qualitative evidence revealed the context-specific mechanisms through which these factors shaped resilience. This divergence may reflect methodological differences rather than conflicting evidence. Quantitative analyses treated determinants as fixed demographic or structural characteristics, while qualitative findings captured the cognitive, behavioral, social, and institutional processes linking them to preparedness, adaptation, and recovery. For instance, education showed no significant association with resilience, but qualitatively, limited education reinforced reliance on short-term coping strategies and constrained long-term adaptation [35]. These findings underscored the added value of mixed-methods synthesis in revealing resilience mechanisms that may not be captured by conventional quantitative indicators alone [36].

In different contexts, flood exposure was unevenly distributed, with coastal communities in Indonesia experiencing increased vulnerability from recurrent flooding, sea level rise, and coastal hazards [40,47]. While flood frequency, depth, and duration increased physical vulnerability through structural damage and livelihood disruptions [40,43], social memory, prior flood experience, and place attachment strengthened preparedness and adaptation [47,48,52]. This indicates that while flood exposure increases physical losses, its effect on preparedness and adaptation depends on broader community and institutional capacities. Moreover, the coexistence of heightened risk awareness with low formal preparedness highlights that awareness alone may be insufficient for adaptive actions, aligning with Protection Motivation Theory [58]. Notably, communities with frequent flooding maintained strong social resilience despite lower assets, suggesting that social capital may compensate for material deficits in high-exposure settings [39].

This divergence suggests that quantitative exposure metrics primarily capture the physical severity of flood events, whereas qualitative evidence explains how social memory, place attachment, collective learning, and institutional support shape adaptive capacity. These findings indicate that flood exposure alone is insufficient to explain resilience outcomes and should be interpreted alongside social and institutional factors. From a practical perspective, disaster risk reduction programmes should therefore consider both hazard exposure and community adaptive capacity when prioritizing preparedness and resilience interventions.

An important consideration is the long-term sustainability of social memory as a resilience mechanism. When knowledge of past floods is transmitted mainly through oral traditions and family experiences, it may gradually decline over three generations, as the last witnesses of past flood events pass away and their experiences are not formally preserved [48,52,53]. This may reduce the continuity of experiential knowledge, particularly where the return period of major flood events exceeds living memory. Formalizing these traces of memory through permanent flood-marker signage, digitized community archives, and ICT-based knowledge platforms may help preserve resilience-related knowledge across generations while complementing, rather than replacing, oral transmission and community-based knowledge practices [52,59].

As another component of the social subsystem, socioeconomic resources supported preparedness, adaptation, and recovery, whereas poverty, unstable livelihoods, indebtedness, and poor housing increased vulnerability and limited long-term recovery [35,39,43], highlighting the role of financial capital in reducing disaster impacts and facilitating recovery. Conversely, communities experiencing poverty, unstable livelihoods, indebtedness, and poor housing conditions appeared more vulnerable to flood impacts and faced greater barriers to recovery, potentially reinforcing existing socioeconomic inequalities [2]. Qualitative evidence further indicated that unstable daily-wage employment, debt burden, and informal livelihoods prevent investment in safer housing and long-term adaptation, reinforcing the cycles of vulnerability over time. In addition, marginalized communities with economic dependency and limited livelihood diversification were constrained to reliance on small-scale, self-help coping strategies [17], a pattern corroborated by quantitative evidence linking informal employment to reduced adaptive capacity [43]. These findings suggest that resilience interventions that focus solely on emergency coping without addressing livelihood security and socioeconomic inequalities may have limited long-term effectiveness.

Within the governance and collective-action subsystem, social capital, community cohesion, and social support consistently functioned as central pillars of flood resilience in Southeast Asia. Quantitative measures showed social capital as the strongest predictor of flood preparedness [45], and the highest-scoring adaptive capacity component [38], while community participation was correlated with social cohesion [46], and active engagement significantly improved resilience outcomes [39]. This finding aligns with global evidence on bonding and bridging social capital as strong recovery predictors [60]. The consistency across Indonesia, Thailand, Malaysia, Myanmar, and Cambodia suggests that social capital represents a culturally robust resilience mechanism in the region. Furthermore, culturally embedded practices like gotong royong and indigenous knowledge systems represent overlooked resilience resources [47,50,53], while technology-mediated collective action in Thailand extends traditional social capital into digital domains [51]. However, community-led responses alone are insufficient for long-term resilience without institutional support [49].

The ecological and physical subsystem comprises environmental and physical determinants, which acted as interconnected structural conditions shaping community flood resilience. Evidence consistently reported that land-use change, degraded ecosystem conditions, substandard infrastructure, and physical capital deficits increased flood sensitivity and vulnerability and decreased communities’ adaptive capacity [35,42,43,49]. Limited access to essential resources, including safe water and reliable energy supply, further weakened resilience. Persistent deficits in natural and physical capital exhibit that community resilience requires long-term investment [35]. These findings align with the IPCC Sixth Assessment Report, which identifies exposure patterns, infrastructure conditions, and adaptive capacity as major drivers of flood risk, particularly among disadvantaged riverine and coastal populations [61,62]. Qualitative evidence further illustrated that fragmented infrastructure and inadequate maintenance often resulted in short-term adaptation rather than sustained resilience, underscoring that community-based adaptation is insufficient without coordinated investment in ecosystem protection, land-use planning, and resilient infrastructure systems [47,48,49].

Psychological determinants functioned as cross-cutting mechanisms within the social subsystem by influencing how structural resources and hazard awareness were translated into adaptive behaviour. The prominence of preparedness knowledge, risk perception, and perceived consequences supports Protection Motivation Theory, which proposes that protective behaviors are more likely when individuals perceive threats and possess confidence in their ability to respond effectively [58]. Conversely, anxiety and emotional distress tend to inhibit preparedness and reduce engagement in adaptive behaviors, particularly when coping resources are limited [63]. This pattern was particularly evident among older populations, where fear of self-evacuation and low confidence in emergency systems reinforced physical and social vulnerabilities [44]. Qualitative findings demonstrated that risk awareness alone was insufficient to promote proactive adaptation when place attachment and socioeconomic dependency restricted behavioral choices, highlighting that psychological responses are embedded within broader social and material contexts [47,63].

This pattern reflects the well-documented risk perception paradox, in which heightened threat awareness does not necessarily translate into protective action. Protection Motivation Theory suggests that this gap may result from low perceived response efficacy and coping appraisal [58]. Our synthesis further indicates that persistent structural deficits, including inadequate drainage, fragile utilities, and repeated infrastructure failure, may reduce perceived response efficacy and contribute to learned helplessness [64], whereby recurrent exposure to unaddressed hazards weakens confidence in the value of protective action despite high-risk awareness [58,61]. Therefore, strengthening trust, self-efficacy, social connectedness, and ICT-supported communication may therefore enhance long-term community resilience [39,41,51].

Collectively, these findings demonstrate that community flood resilience is not determined by any single factor but emerges from dynamic interactions among the social, governance/collective-action, and ecological/physical subsystems of the SES framework. Sociodemographic and socioeconomic determinants shape baseline vulnerability and access to adaptive resources, while sociocultural determinants strengthen social capital, collective action, and community governance that facilitate coordinated preparedness and recovery. Environmental and physical determinants influence exposure, infrastructure, and ecological conditions that either enable or constrain resilience, whereas psychological determinants operate across these subsystems by influencing whether hazard awareness and available resources are translated into adaptive and protective behaviours.

Through the lens of the Sendai Framework for Disaster Risk Reduction 2015–2030 [10], these domains align with its four priorities for action: sociodemographic and psychological determinants underpin understandings of disaster risk; sociocultural determinants strengthen governance and collective action; environmental and physical determinants inform investment in resilience infrastructure and ecosystems; and socioeconomic determinants, together with flood exposure, shape preparedness for effective response and recovery. This alignment indicates that strengthening community flood resilience requires integrated action across all four priorities rather than addressing individual risk factors in isolation. The integrated systems perspective highlights the importance of coordinated public health and disaster risk reduction strategies to reduce vulnerability and promote sustainable flood resilience in Southeast Asian communities.

### 4.3. Population Vulnerability and Public Health Challenges

Flood vulnerability reflects interconnected social, economic, and environmental inequalities with multidimensional consequences for health and well-being. Flooding poses a wide range of infectious, mental, and physical health effects, as well as livelihood and economic disruptions that intensify vulnerability over time [65]. Across studies, vulnerability extended beyond flood exposure and reflected disparities in access to essential resources and healthcare, arising from compounding weaknesses across the social, governance and collective-action, and ecological and physical subsystems of the SES framework rather than from any single deficit. These findings align with social determinants of health frameworks, emphasizing that pre-existing disadvantage influences preparedness, adaptive capacity, and post-disaster recovery [61,66].

Regarding age, older residents may be at higher risk through the social subsystem, where their mobility dependence, chronic health conditions, and constrained family and institutional support networks limit emergency response capacity and create barriers to evacuation and recovery [35,39,43,44]. Similarly, low-income households face compounding deficits across socioeconomic dimensions of the social subsystem, including reduced medical access, inadequate preparedness supplies, and compromised hygiene, with debt cycles preventing recovery investment [17,39,43,44,46]. Female-headed households also experience unequal care burden, power structures, and social isolation that affect psychological well-being and reduce adaptive capacity, compounded by their limited representation within the governance and collective-action subsystem [35,44].

Moreover, informal settlement residents experienced vulnerability through interacting SES subsystems, where limited participation in formal governance and inadequate water, sanitation, and shelter infrastructure increased environmental health risks [17,47]. However, their reliance on gotong royong (mutual cooperation) demonstrates how collective action can partially compensate for institutional exclusion [47]. Rural agricultural communities similarly experienced livelihood disruption, food insecurity, and psychological trauma from landscape transformation [39,52]. Ultimately, reducing flood vulnerability requires strengthening governance and collective action, resilient infrastructure, and adaptive capacity across all three SES subsystems, consistent with the Sendai Framework’s priorities for disaster risk reduction and resilience building [10].

### 4.4. Policy Implications and Recommendations

Policy recommendations are prioritized according to the strength and consistency of the underlying evidence and organized according to the three SES subsystems through which they operate. Within the governance and collective-action subsystem, the strongest evidence, derived from the sociocultural determinants (13 of 18 studies), consistently identified social capital, community cohesion, and indigenous cooperative practices, such as gotong royong, as key contributors to community flood resilience. These findings support complementing top-down disaster risk reduction strategies with knowledge co-production, in which village or sub-district level authorities and community leaders co-design emergency plans, warning signage, evacuation routes, and preparedness activities by integrating local knowledge, cultural practices, and existing community capacities into formal disaster risk reduction planning. Because governance structures vary substantially across Southeast Asia, from Indonesia’s decentralized village governance to more centrally coordinated systems in parts of Cambodia and Vietnam, this co-production approach requires adaptation to local administrative capacity rather than uniform application.

Within the social subsystem, moderate evidence from the socioeconomic domain suggests that community-based approaches should be complemented by targeted investments in livelihood support, safer housing, and basic infrastructure, particularly for vulnerable populations living in informal or flood-prone settlements. However, these investments are often constrained by political economy factors within the governance and social dimensions of the SES framework. In Informal settlements, insecure land tenure can discourage both household investment in housing upgrades and government investment in permanent infrastructure, while short political and funding cycles may reduce sustained commitment to long-term resilience infrastructure over visible, short-term relief measures. Where quantitative and qualitative findings diverged, such as for education, locally adapted interventions may be more appropriate than uniform policy approaches.

Within the ecological and physical subsystem shaping communities’ exposure to flooding, priorities differ by time horizon. In the short term, strengthening community-based early warning systems, age- and gender-responsive evacuation planning, and the involvement of community health workers and local leaders may enhance preparedness at low cost and political friction. Longer-term priorities include sustained investment in drainage and utility infrastructure, ecosystem protection, land-use planning reform, and the formal integration of indigenous knowledge into disaster risk reduction policies. However, these measures require substantial financial commitments and may be constrained by competing budget priorities and broader political economy factors, underscoring the need for dedicated multi-year financing mechanisms rather than reliance on post-disaster relief budgets.

Across all three subsystems, this review highlights that the populations most affected by flooding, including the elderly, women, low-income households, and residents of informal settlements, face the greatest flood exposure and health risks while having the least influence over disaster planning and resource allocation. This imbalance reflects not only resource scarcity but also structural exclusion from decision-making processes, highlighting the political and ecological dimension of public health challenges. Addressing these inequities requires deliberate representation of vulnerable groups within local authorities. Therefore, strengthening participatory governance and community representation in local disaster risk reduction planning may help ensure that resilience investments better reflect the needs of vulnerable populations.

Future research should prioritize longitudinal designs and standardized measurement tools. Country-comparative studies are also needed to distinguish context-specific determinants from generalizable ones across Southeast Asian countries. Moreover, greater attention to gender-disaggregated analyses and quantitative assessment of indigenous knowledge, cultural practices, and social memory is needed to better understand their contribution to community flood resilience.

### 4.5. Strengths and Limitations

This review has several strengths. Registration with PROSPERO and adherence to PRISMA 2020 guidelines ensured transparency and reproducibility. The comprehensive five-database search, supplemented by Google Scholar, maximized literature recall. In addition, dual independent screening and quality assessment minimized reviewer bias, while convergent integrated synthesis preserved methodological integrity. Finally, the inclusion of quantitative, qualitative, and mixed-methods studies across six Southeast Asian countries provides contextually grounded evidence.

However, limitations are acknowledged. Firstly, the restriction to English-language publications may have excluded relevant studies published in regional languages or less-indexed sources, introducing language bias. This may have contributed to the predominance of Indonesian studies (50% of included studies) and the limited evidence identified from Myanmar and Vietnam. Future systematic reviews should consider multilingual search strategies or collaboration with regional researchers to reduce this potential bias.

Secondly, the evidence base was geographically concentrated in Indonesia and Thailand, with no eligible studies from Laos, the Philippines, Singapore, Brunei, or Timor-Leste. Consequently, the findings are more representative of Island Southeast Asia and Thailand than the entire Southeast Asian region. This geographic concentration may reflect differences in research capacity, flood exposure and research attention, as well as underrepresentation of non-English or less-indexed literature. Therefore, the findings should be generalized to underrepresented countries with caution. Thirdly, the predominance of cross-sectional designs constrains causal inference, whereas self-report bias and exclusion of grey literature are additional limitations. Finally, heterogeneity in resilience definitions, operational measures, theoretical frameworks, and study populations limited direct comparison across studies, precluded meta-analysis, and necessitated narrative synthesis.

## 5. Conclusions

This systematic review provides a comprehensive synthesis of determinants influencing community flood resilience in Southeast Asia based on 18 high-quality studies across Indonesia, Thailand, Malaysia, Vietnam, Myanmar, and Cambodia. The findings indicate that community flood resilience is shaped by dynamic interactions across sociodemographic, flood exposure, socioeconomic, sociocultural, environmental, physical, and psychological domains rather than by isolated factors. Synthesized evidence supports a multidimensional understanding of resilience in which structural conditions, community processes, and cognitive-psychological capacities collectively influence adaptive capacity and recovery. These findings further highlight that community flood resilience extends beyond disaster preparedness and represents a broader public health challenge shaped by social determinants of health.

The review also identified disproportionate vulnerability among older adults, low-income households, women in female-headed households, informal settlement residents, and rural agricultural communities, underscoring the need for more equitable and inclusive disaster risk reduction approaches. Building long-term flood resilience in Southeast Asia requires integrated strategies that address underlying inequalities, strengthen community and infrastructure capacity, incorporate indigenous knowledge, and promote adaptive and empowerment-oriented responses. Although the concentration of evidence in Indonesia may limit regional generalizability, the findings highlight the importance of coordinated action across public health, disaster risk reduction, and development sectors to support sustainable adaptation in flood-prone communities.

## Figures and Tables

**Table 3:** Vulnerable populations and related public health challenges in flood-affected communities.

Population Group	Vulnerability Characteristics	Public Health Challenges	Implications for Flood Resilience	Supporting Studies
Older adults	Physical limitations; dependence on mobility aids; chronic illness/poor health conditions; loneliness; low education.	Evacuation difficulties; mobility limitation during emergencies; inadequate emergency health care access; worsening physical health; psychological stress; limited continuity of care.	Lower adaptive capacity and reduced resilience without accessible healthcare, institutional support, and family networks.	[35,43,44]
Low-income households	Limited financial resources; low education; informal employment; high debt burden; reduced credit accessibility; poor housing quality; informal housing in flood-prone areas.	Reduced access to healthcare; inadequate preparedness supplies and safe housing; inability to afford recovery; compromised hygiene and sanitation.	Increased flood vulnerability; reduced social resilience without financial support; lower preparedness, adaptation and recovery after flood events.	[17,39,43,44,45,46]
Women	Unequal resource access; caregiving burdens; limited independent financial assets; lesser community voice; social isolation.	Stress from social isolation and care duties, limiting flood evacuation and recovery; inadequate institutional support structures for women.	Lower adaptive capacity; marital status (widowhood) as a critical vulnerability indicator; lack of disaster management representation.	[35,39,41,44]
Informal settlements/Slum households	Lack of secure land tenure; limited drainage, clean water and garbage disposal systems; limited financial capital; inadequate housing infrastructure; marginalization from formal emergency response; high population density.	Lack of clean water/sanitation; increased risk of infectious diseases; limited access to shelter and medical aid during flood events; chronic psychosocial stress; garbage accumulation from extended inundation; environmental health decline.	Reliance on communal self-organization; reduced resilience capacity; need for community-based adaptation (CBA); gotong royong (mutual cooperation) functioned as social capital despite financial limitation.	[17,47,48,49]
Rural agricultural communities	Lack of protective mitigation structures; limited income-sharing opportunities; restricted land and water resource accessibility; dependence on specific ecological services; shifting hydrological patterns.	Livelihoods disruptions, food and water insecurity; poor access to healthcare and essential services; poor physical health; psychological trauma from landscape transformation; loss of cultural identity.	Limited adaptation by landscape changes; prior flood experience served as a foundation for community-based resilience building; need for incorporating social memory and local knowledge into flood policy.	[39,52]

## Data Availability

The original contributions presented in this study are included in the article/supplementary material. Further inquiries can be directed to the corresponding author.

## Supplementary Materials

The following supporting information can be downloaded at: https://www.mdpi.com/article/10.3390/ijerph23081013/s1, Table S1: Convergence synthesis of determinants influencing community flood resilience by domain; Table S2: PRISMA 2020 checklist; Table S3: PRISMA 2020 for abstracts checklist; Table S4: Articles excluded in full-text review and reasons for exclusion [67,68,69,70,71,72,73,74,75,76,77,78,79,80,81,82,83,84,85,86,87,88,89,90,91,92,93,94,95,96,97].

## Appendix A

**Table A1:** Critical appraisal results for six qualitative studies and qualitative strands of three mixed-method studies using quality appraisal tools [34].

First Author (Year)	Q1	Q2	Q3	Q4	Q5	Q6	Q7	Q8	Q9	Q10	Total Score	Quality Appraisal (%)
Aksa, F.I., et al. (2022) [47]	2	2	2	1	1	2	1	0	2	0	13/20	65.0%
Priyanti, R.P., et al. (2019) [48]	2	2	2	2	1	2	2	1	2	0	16/20	80.0%
Marfai, M.A., et al. (2015) [49]	2	2	2	2	2	2	1	0	2	0	15/20	75.0%
Dwinantoaji, H., et al. (2020) [50]	2	2	2	1	2	2	2	2	2	1	18/20	90.0%
Leong, C.M.L., et al. (2015) [51]	2	2	2	2	1	2	2	1	2	1	17/20	85.0%
Tran, T.A., et al. (2022) [52]	2	2	2	2	2	2	2	1	2	1	18/20	90.0%
Yang, Y., et al. (2024) [35]	2	2	2	2	1	1	1	1	2	1	15/20	75.0%
Yang, J., & Andriesse, E. (2021) [17]	2	2	2	2	1	2	1	1	2	1	16/20	80.0%
Markolinda et al. (2025) [53]	2	2	2	1	1	1	1	1	2	0	13/20	65.0%

Note: Yes = 2; Partial = 1; and No = 0. Total possible score = 20, % = total obtained score/20 × 100. High quality ≥ 75%, Moderate quality = 50–74%, Low quality < 50%.

**Table A2:** Critical appraisal results for nine quantitative studies and quantitative strands of three mixed-method studies using quality appraisal tools [34].

First Author (Year)	Q1	Q2	Q3	Q4	Q5	Q6	Q7	Q8	Q9	Q10	Q11	Q12	Q13	Q14	Total Score	Quality Appraisal (%)
Kumaresen, M., et al. (2025) [38]	2	2	1	2	N/A	N/A	N/A	2	1	2	2	1	2	2	19/22	86%
Lwin, K.K., et al. (2020) [39]	2	2	2	2	N/A	N/A	N/A	2	2	2	2	1	2	2	21/22	95.5%
Isa, M., et al. (2018) [40]	2	2	1	1	N/A	N/A	N/A	2	1	2	1	1	2	2	17/22	77.3%
Thapa, S., et al. (2025) [41]	2	2	2	2	N/A	N/A	N/A	2	2	2	2	2	2	2	22/22	100%
Nugraheni, I.L., et al. (2022) [42]	2	2	2	2	N/A	N/A	N/A	2	2	2	2	1	2	2	21/22	95.5%
Mruksirisuk, P., et al. (2023) [43]	2	2	2	1	N/A	N/A	N/A	2	2	2	2	1	2	2	20/22	90.9%
Saputra, H., et al. (2025) [44]	2	2	2	2	N/A	N/A	N/A	2	2	2	1	1	2	2	20/22	90.9%
Ningtias, Y.R., et al. (2025) [45]	2	2	2	2	N/A	N/A	N/A	2	1	2	2	1	2	1	19/22	86.4%
Ludin, S.M., et al. (2019) [46]	2	2	2	2	N/A	N/A	N/A	2	2	2	2	1	2	1	20/22	90.9%
Yang Y. et al. (2024) [35]	2	2	1	2	N/A	N/A	N/A	2	1	1	0	1	1	1	14/22	63.64%
Yang, J., & Andriesse, E. (2021) [17]	2	2	2	2	N/A	N/A	N/A	2	1	1	0	1	1	1	15/22	68.2%
Markolinda et al. (2025) [53]	2	2	2	2	N/A	N/A	N/A	1	2	1	0	0	1	1	14/22	63.64%

Note: Yes = 2; Partial = 1; and No = 0; N/A = not applicable. Total possible score = 22, % = total obtained score/22 × 100. High quality ≥ 75%, Moderate quality = 50–74%, Low quality < 50%.
