# Supplementary material for: Exploring Determinants of Community Flood Resilience in Southeast Asia: A Systematic Review
Source: Int J Environ Res Public Health. 2026 Aug 2;23(8):1013. doi: 10.3390/ijerph23081013 (PMC13512627; doi:10.3390/ijerph23081013)
Supplement: Supplementary file 1 [file ijerph-23-01013-s001.zip › ijerph-4422025-supplementary.pdf]

Supplementary materials

Exploring determinants of community flood resilience in Southeast Asia: a systematic review

Nan Ei Moh Moh Kyi, Muhammad Naeem Rashid , Shun Lae Lae Aung , Tipsuda Pintakham , and Anurak Wongta

The Supplementary Material provides information on PRISMA reporting checklists (Tables S1-2).

A full list of the supplementary tables is provided below.

Table S1. Convergence synthesis of determinants influencing community flood resilience by domain.

Table S2. PRISMA 2020 checklist.

Table S3. PRISMA 2020 for abstracts checklist.

Table S4. Articles excluded in full-text review and reasons for exclusion.

Table S1. Convergence synthesis of determinants influencing community flood resilience by domain.

Determinants by domain	Quantitative Evidence	Qualitative Evidence	Synthesized interpretation	Supporting studies
**Socio-demographic**<br><br>Age<br>Household size<br>Proportion of household dependents<br>Education<br>Health status	▪ **Age:** distinct resilience barrier; positively correlated with physical limitations during crises (*r*=0.68, p<0.001), but no evidence on age-related adaptive mechanisms.<br>▪ **Household size: highest** sensitivity indicator score (0.901), confirming elevated household vulnerability index.<br>▪ **A high proportion of household dependents (**elderly/children): significantly increased vulnerability indices (p<0.05).<br>▪ **Health status:** critical component of resilience driver, with health condition and physical mobility loading at 0.168 and 0.171 on PC2.	▪ **The elderly** were identified as a vulnerable group requiring targeted support, but no evidence on age-related adaptive mechanisms.<br>▪ **Larger households** constrained adaptive capacity and increased recovery challenges.<br>▪ **High concentrations of dependents** presented immediate physical evacuation and recovery challenges.<br>▪ **Education** served as an enabler of adaptive capacity through training and knowledge acquisition, but low education constrained adaptation and reinforced short-term coping strategies.<br>▪ **Health vulnerability** emerged as a flood exposure multiplier through injury, disease burden, disability, and basic coping practices.	▪ Quantitative and qualitative evidence identify older age as a vulnerability factor, though qualitative insight remains limited regarding age-specific adaptation pathways and resilience mechanisms.<br>▪ Larger households and high concentration of dependents increase flood vulnerability through compounded caregiving and resource demands during sudden-onset evacuations.<br>▪ While quantitative findings showed no significant association between education and resilience capacity, qualitative evidence indicated that low education posed path-dependent coping, reduced risk awareness, and limited capacity to pursue transformative adaptation strategies.<br>▪ Complementary evidence: Quantitative component loadings demonstrate the structural necessity of health assets, while qualitative data highlights how disease burdens directly deplete community savings.	Saputra, H., et al. (2025) [44]<br>Mruksirisuk, P., et al. (2023) [43]<br>Yang, Y., et al. (2024)[35]<br>Priyanti et al. (2022)[48]<br>Yang & Andriesse (2021)[17]

Table S1. Convergence synthesis of determinants influencing community flood resilience by domain (Continued.)

Determinants by domain	Quantitative Evidence	Qualitative Evidence	Synthesized interpretation	Supporting studies
Flood exposure<br><br>Flood frequency, depth, duration<br>Riverine/coastal proximity<br>Prior flood experience	▪ **Physical flood exposure** (flood frequency, depth, duration, riverine/coastal proximity) positively predicted resilience (β=0.354, p<0.05); damage and losses exerted strongest impact (RI=0.94 vs 0.93), followed by casualties (RI=0.69).<br>▪ **Prolonged depth and duration** correlated with structural damage. (r = 0.741, p < 0.01)<br>▪ High-exposure areas reported higher risk perception (WAI=0.90) but lower asset capital (WAI≤0.68); social resilience and adaptive capacity remained strong across exposure levels (WAI≥0.68).<br>▪ **Proximity to water bodies** predicted vulnerability geographically (β= 0.312, p < 0.001), and contributed to flood exposure scores (Indicator Score [IS]=0.721)	▪ Repeated flood exposure built social memory, connecting past experience to present adaptation and catalyzing shifts from incremental to transformative adaptation after disruptive events.<br>▪ Protracted standing water directly destroys local road networks, halting physical access and emergency distribution.<br>▪ Despite recurrent past flood experience, flood preparedness remained low, moderated by social cognition, limited community capacity and location attachment rather than exposure alone.<br>▪ Although cumulative flood experience was associated with high-risk awareness, it was inversely linked to formal preparedness behaviors such as relocation.	▪ Quantitative and qualitative evidence converge on the structural severity of flood exposure: statistical damage coefficients match qualitative narratives.<br>▪ Evidence diverges on behavioral and cognitive pathways: quantitative findings associate exposure with elevated risk perception while qualitative findings indicate that preparedness depends on social memory, place attachment, and community capacity rather than exposure frequency alone.<br>▪ Complementary: transformative adaptation emerges where cumulative experience intersects with social cognition, not from exposure itself.	Lwin, K. K.,et al. (2020) [39]<br>Isa, M., et al. (2018) [40]<br>Aksa, F. I., et al. (2022) [47]<br>Priyanti, R. P., et al. (2019) [48]<br>Marfai, M.A., (2015) [49]<br>Tran, T. A., et al. (2022) [52]

Table S1. Convergence synthesis of determinants influencing community flood resilience by domain (Continued.)

Determinants by domain	Quantitative Evidence	Qualitative Evidence	Synthesized interpretation	Supporting studies
**Socio-economic**<br><br>Income level<br>Debt<br>Financial capital<br>Livelihood diversification/ dependence<br>Employment status<br>Housing condition	▪ **Low-income** and **debt** increased exposure (index=0.531) and sensitivity (index=0.633); household income constrained adaptive capacity (index=0.503).<br>▪ **Higher income** was associated with greater social resilience capacity (Chi-square = 31.62, $p < 0.001$).<br>▪ **Informal employment:** baseline characteristics linked to low adaptive capacity scores ($p < 0.05$).	▪ **Financial capital**: low income, limited financial resources, lack of income diversification were major barriers to community flood resilience, especially among poor households.<br>▪ **Informal livelihoods** entailed unstable income, limited savings, debt, lack of safety nets, and post-flood job loss.<br>▪ **Economic dependency and path-dependent coping** increased cumulative vulnerability and maladaptive coping over time.<br>▪ **Limited livelihood diversification/dependence,** and economic opportunities constrained adaptation options, and increased damage and losses, reinforced reliance on small-scale, self-help coping.<br>▪ **Poor housing conditions compounded** vulnerability; low-income households relied on short-term coping and communal resources.	▪ Both evidence converge on low income, debt, weak financial capital, limited livelihood diversification as constraints on adaptive capacity and amplifiers of flood sensitivity.<br>▪ Quantitative indices map exposure and sensitivity thresholds; qualitative findings explain how daily-wage instability and debt generate cumulative, path-dependent vulnerability, which prevents housing investment and channels households toward small-scale coping.<br>▪ Complementary: quantitative metrics capture structural deficits, while qualitative accounts demonstrate communal resources as compensatory, but insufficient adaptation.	Andriesse, E. (2021) [17]<br>Yang Y. et al. (2024) [35]<br>Lwin, K. K.,et al. (2020) [39]<br>Mruksirisuk, P., et al. (2023) [43]<br>Yang, J., &<br>Aksa, F. I., et al. (2022) [47]<br>Tran, T. A., et al. (2022) [52]

Table S1. Convergence synthesis of determinants influencing community flood resilience by domain (Continued.)

Determinants by domain	Quantitative Evidence	Qualitative Evidence	Synthesized interpretation	Supporting studies
**Socio-cultural factors**<br><br>Social capital<br>Community cohesion<br>Collective action<br>Community participation<br>Mutual cooperation (gotong royong)<br>Social support<br>Indigenous knowledge<br>Social media<br>Cultural practices	▪ **Social capital** predicted flood preparedness (β=0.659, p<0.01), and scored highest for adaptive capacity (Mean index=7.86).<br>▪ **Social cohesion** linked to adaptive capacity across exposure levels (WAI≥0.68).<br>▪ **Collective action**, trust, social networks enhanced community resilience and social cohesion (p<0.01).<br>▪ **Community participation** correlated with social cohesion (*r*=0.529, p<0.001), and active community engagement improved outcomes (p<0.001).<br>▪ **Human/ social capital (participation, coordination, networks)** strengthened resilience longitudinally.<br>▪ **Social support** also predicted household flood preparedness (*β*=0.134, p<0.001)	▪ **Social capital**, leadership, stakeholder coordination, and family/community attachment strengthened preparedness and reduced vulnerability<br>▪ **Mutual cooperation (gotong royong),** organizational support, and community participation mobilized community response capacities.<br>▪ **ICT and social media** facilitated empowerment through information access and collective action.<br>▪ **Indigenous knowledge and cultural practices** enabled transformative adaptation.	▪ Both evidence consistently identified social capital, cohesion and support as primary resilience enablers across Southeast Asian settings.<br>▪ Quantitative metrics quantify predictive strength, while qualitative evidence reveals activation mechanisms (mutual cooperation, cultural practices, ICT-driven mobilization) that transform latent social capital into protective action during flood crises.<br>▪ Quantitative frameworks measure social capital as generic network capacity; qualitative evidence specifies how indigenous knowledge and cultural practices enable transformative adaptation distinct from formal institutional responses.	Yang Y. et al. (2024) [35]<br>Kumaresen M, et al. (2025) [38]<br>Lwin, K. K.,et al. (2020) [39]<br>Thapa, S., et al. (2025) [41]<br>Nugraheni, I. L., et al. (2022) [42]<br>Ningtias, Y.R., et al. (2025) [45]<br>Ludin, S.M., et al. (2019) [46]<br>Aksa, F.I. et al. (2022) [47]<br>Priyanti, R. P., et al. (2019) [48]<br>Marfai, M. A., et al. (2015) [49]<br>Dwinantoaji, H., et al. (2020) [50]<br>Leong, C. M. L., et al. (2015) [51]<br>Tran, T. A., et al. (2022) [52]<br>Markolinda et al. (2025) [53]

Table S1. Convergence synthesis of determinants influencing community flood resilience by domain (Continued.)

Determinants by domain	Quantitative Evidence	Qualitative Evidence	▪ Synthesized interpretation	Supporting studies
**Environmental factors**<br><br>Land-use Infrastructure quality Natural capital	▪ Confirmatory Factor Analysis (CFA) validated **land-use patterns** (factor loading = 0.704) and **substandard drainage systems** (factor loading = 0.789) as vulnerability drivers.<br>▪ **Land-use** impacts on flood resilience were validated with CLUE-S model (κ = 0.581).<br>▪ **Natural capital (ecosystem condition)** emerged as a critical vulnerability driver with no improvement over time. (FRMC s=33/100).	▪ Urban land-use change and coastal subsidence exacerbated the frequency of tidal flooding, overwhelming traditional adaptation.<br>▪ Infrastructure quality (poor drainage, waste/sanitation quality, and maintenance) contributed to small-scale, uncoordinated adaptation and amplified flood exposure.<br>▪ Ecosystem-based adaptation was one mechanism by which social memory translated into resilience-building responses.	▪ Land-use change, degraded drainage infrastructure, and ecosystem decline constitute structural drivers of flood vulnerability in both evidence streams.<br>▪ Quantitative factor loadings quantify systematic vulnerability, while qualitative evidence further enriches how environmental stressors convert predictable seasonal floods into frequent, unmanageable tidal inundations that overwhelm traditional coping thresholds.<br>▪ Quantitative natural capital scores stagnate, while qualitative evidence identifies ecosystem-based adaptation as a pathway translating social memory into resilience-building responses.	Yang Y. et al. (2024) [35]<br>Nugraheni, I. L., et al. (2022) [42]<br>Mruksirisuk, P., et al. (2023) [43]<br>Aksa, F. I., et al. (2022) [47]<br>Marfai, M. A., et al. (2015) [49]<br>Tran, T. A., et al. (2022) [52]

Table S1. Convergence synthesis of determinants influencing community flood resilience by domain (Continued.)

Determinants by domain	Quantitative Evidence	Qualitative Evidence	Synthesized interpretation	Supporting studies
**Physical factors**<br><br>Building quality<br>Utility disruptions<br>Drainage adequacy<br>Physical capital	▪ **Building quality and severe utility disruptions** were key flood sensitivity drivers (sensitivity index=0.611 vs 0.747)<br><br>▪ **Adequate drainage system** was lowest-rated preparedness item (M=3.62), indicating infrastructural deficiency.<br><br>▪ **Drainage infrastructure** scored moderately for adaptive capacity (converted scores=0.559-0.571), while warning and assistance measures scored lowest, denoting strongest adaptive capacity (converted score=0.257).<br><br>▪ **Physical capital** (infrastructure, access to safe water and energy supply) deficits (17–33% below standard) limited flood resilience, while financial stagnation and low natural capital (33/100) further constrained adaptive capacity.	▪ **Poor drainage, waste/sanitation, weak infrastructure quality and maintenance** contributed to small-scale, uncoordinated adaptation and amplified flood exposure.<br><br>▪ Communities relied more on human-centered early warning systems & mobilization.	▪ Convergence on infrastructure deficits: Building quality, utility disruptions, drainage inadequacy heighten flood sensitivity and depress adaptation.<br><br>▪ Quantitative indices measure deficit severity, while qualitative evidence reveals how communities compensate through human-centered early warning systems and collective mobilization.<br><br>▪ Divergent: Quantitative scores indicate persistent infrastructural deficiency; qualitative findings demonstrate that community-led adaptations remain insufficient for sustained resilience	Yang Y. et al. (2024) [35]<br>Kumaresen M, et al. (2025) [38]<br>Mruksirisuk, P., et al. (2023) [43]<br>Priyanti, R. P., et al. (2019) [48]<br>Marfai, M. A., et al. (2015) [49]

Table S1. Convergence synthesis of determinants influencing community flood resilience by domain (Continued.)

Determinants by domain	Quantitative Evidence	Qualitative Evidence	Synthesized interpretation	Supporting studies
**Psychological factors**<br><br>Preparedness knowledge<br>Flood risk perception<br>Perceived consequences<br>Anxiety<br>Emotional confidence<br>Fear<br>Self-efficacy<br>Trust	▪ **Preparedness knowledge** was the strongest predictor of proactive adaptation (β=0.539), followed by perceived risk (β = 0.202), and perceived consequences (β = 0.193) at p<0.001.<br><br>▪ **Anxiety** was negatively associated with preparedness (β=−0.124, p=0.010)<br>▪ **Low confidence** in emergency rescue systems (β = -0.174) and **self-evacuation fear** (β = -0.245) significantly reduced elderly resilience scores at p < 0.01).<br><br>▪ Higher **trus**t in officials and emergency services (Chi-square = 9.84, p < 0.01), together with flood risk awareness and actionable flood protection knowledge (p<0.01), were significant determinants of high social resilience.	▪ Despite high flood risk awareness, communities stayed due to limited relocation options, proximity to birthplace/kinship, and economic dependence, indicating that risk perception alone did not lead to adaptation.<br><br>▪ **Risk knowledge and perception** were components of the five themes shaping community perception of flood preparedness.<br><br>▪ Negative past exposure combined with bad infrastructure generates a sense of powerlessness and community passivity.<br><br>▪ Real-time communication **(ICT/social media)** built self-efficacy, converting shared risk into organized community action.	▪ Convergent evidence confirms that knowledge, perception, and trust promote preparedness, whereas anxiety and fear reduce it.<br><br>▪ Quantitative models identify risk perception as a direct predictor, while qualitative evidence reveals that awareness alone is insufficient when place attachment and economic dependence restrict choices.<br><br>▪ Complementary: Coefficients identify trust and knowledge as drivers, while qualitative evidence explains how trust erodes through negative experiences, but rebuilds through ICT-mediated self-efficacy and collective action.	Lwin, K. K.,et al. (2020) [39]<br>Thapa, S., et al. (2025) [41]<br>Saputra, H., et al. (2025) [44]<br>Aksa, F. I., et al. (2022) [47]<br>Priyanti, R. P., et al. (2019) [48]<br>Leong, C. M. L., et al. (2015) [51]

Abbreviations: *r* = Pearson correlation coefficient; RI= resilience indicator***;*** WAI= weighted average index; β = standardized beta coefficient; κ = Cohen's kappa coefficient; FRMC s= flood resilience measurement for communities scores; ICT= information and communication technology; CLUE-S model = The Conversion of Land Use and its Effects at Small regional extent

Table S2. PRISMA 2020 checklist

Section and Topic	Item #	Checklist item	Location where item is reported
**TITLE**			
Title	1	Identify the report as a systematic review.	Page 1
**ABSTRACT**			
Abstract	2	See the PRISMA 2020 for Abstracts checklist.	Page 2
**INTRODUCTION**			
Rationale	3	Describe the rationale for the review in the context of existing knowledge.	Page 3-4
Objectives	4	Provide an explicit statement of the objective(s) or question(s) the review addresses.	Page 4
**METHODS**			
Eligibility criteria	5	Specify the inclusion and exclusion criteria for the review and how studies were grouped for the syntheses.	Page 4-5
Information sources	6	Specify all databases, registers, websites, organisations, reference lists and other sources searched or consulted to identify studies. Specify the date when each source was last searched or consulted.	Page 5
Search strategy	7	Present the full search strategies for all databases, registers and websites, including any filters and limits used.	Page 5
Selection process	8	Specify the methods used to decide whether a study met the inclusion criteria of the review, including how many reviewers screened each record and each report retrieved, whether they worked independently, and if applicable, details of automation tools used in the process.	Page 5
Data collection process	9	Specify the methods used to collect data from reports, including how many reviewers collected data from each report, whether they worked independently, any processes for obtaining or confirming data from study investigators, and if applicable, details of automation tools used in the process.	Page 5
Data items	10a	List and define all outcomes for which data were sought. Specify whether all results that were compatible with each outcome domain in each study were sought (e.g. for all measures, time points, analyses), and if not, the methods used to decide which results to collect.	Page 6
	10b	List and define all other variables for which data were sought (e.g. participant and intervention characteristics, funding sources). Describe any assumptions made about any missing or unclear information.	Page 6
Study risk of bias assessment	11	Specify the methods used to assess risk of bias in the included studies, including details of the tool(s) used, how many reviewers assessed each study and whether they worked independently, and if applicable, details of automation tools used in the process.	Page 6
Effect measures	12	Specify for each outcome the effect measure(s) (e.g. risk ratio, mean difference) used in the synthesis or presentation of results.	N/A
Synthesis methods	13a	Describe the processes used to decide which studies were eligible for each synthesis (e.g. tabulating the study intervention characteristics and comparing against the planned groups for each synthesis (item #5)).	Page 6-7
	13b	Describe any methods required to prepare the data for presentation or synthesis, such as handling of missing summary statistics, or data conversions.	N/A
	13c	Describe any methods used to tabulate or visually display results of individual studies and syntheses.	Page 6-7

Section and Topic	Item #	Checklist item	Location where item is reported
	13d	Describe any methods used to synthesize results and provide a rationale for the choice(s). If meta-analysis was performed, describe the model(s), method(s) to identify the presence and extent of statistical heterogeneity, and software package(s) used.	Page 6-7
	13e	Describe any methods used to explore possible causes of heterogeneity among study results (e.g. subgroup analysis, meta-regression).	N/A
	13f	Describe any sensitivity analyses conducted to assess robustness of the synthesized results.	N/A
Reporting bias assessment	14	Describe any methods used to assess risk of bias due to missing results in a synthesis (arising from reporting biases).	N/A
Certainty assessment	15	Describe any methods used to assess certainty (or confidence) in the body of evidence for an outcome.	N/A
**RESULTS**			
Study selection	16a	Describe the results of the search and selection process, from the number of records identified in the search to the number of studies included in the review, ideally using a flow diagram.	Figure 1
	16b	Cite studies that might appear to meet the inclusion criteria, but which were excluded, and explain why they were excluded.	Table S4
Study characteristics	17	Cite each included study and present its characteristics.	Table 1
Risk of bias in studies	18	Present assessments of risk of bias for each included study.	Page 22-23<br>Tables A1-2
Results of individual studies	19	For all outcomes, present, for each study: (a) summary statistics for each group (where appropriate) and (b) an effect estimate and its precision (e.g. confidence/credible interval), ideally using structured tables or plots.	Table S1
Results of syntheses	20a	For each synthesis, briefly summarise the characteristics and risk of bias among contributing studies.	Page 9-12<br>Table 1-2<br>Page 16<br>Table-3<br>Page 22-23<br>Tables A1-2
	20b	Present results of all statistical syntheses conducted. If meta-analysis was done, present for each the summary estimate and its precision (e.g. confidence/credible interval) and measures of statistical heterogeneity. If comparing groups, describe the direction of the effect.	N/A
	20c	Present results of all investigations of possible causes of heterogeneity among study results.	N/A
	20d	Present results of all sensitivity analyses conducted to assess the robustness of the synthesized results.	N/A
Reporting biases	21	Present assessments of risk of bias due to missing results (arising from reporting biases) for each synthesis assessed.	N/A

Section and Topic	Item #	Checklist item	Location where item is reported
Certainty of evidence	22	Present assessments of certainty (or confidence) in the body of evidence for each outcome assessed.	N/A
**DISCUSSION**			
Discussion	23a	Provide a general interpretation of the results in the context of other evidence.	Page 16-20
	23b	Discuss any limitations of the evidence included in the review.	Page 21
	23c	Discuss any limitations of the review processes used.	Page 21
	23d	Discuss implications of the results for practice, policy, and future research.	Page 20-21
**OTHER INFORMATION**			
Registration and protocol	24a	Provide registration information for the review, including register name and registration number, or state that the review was not registered.	Page 4
	24b	Indicate where the review protocol can be accessed, or state that a protocol was not prepared.	Page 4
	24c	Describe and explain any amendments to information provided at registration or in the protocol.	N/A
Support	25	Describe sources of financial or non-financial support for the review, and the role of the funders or sponsors in the review.	N/A
Competing interests	26	Declare any competing interests of review authors.	N/A
Availability of data, code and other materials	27	Report which of the following are publicly available and where they can be found: template data collection forms; data extracted from included studies; data used for all analyses; analytic code; any other materials used in the review.	N/A

Table S3. PRISMA 2020 for abstracts checklist

Section and Topic	Item #	Checklist item	Reported (Yes/No)
**TITLE**			
Title	1	Identify the report as a systematic review.	Yes
**BACKGROUND**			
Objectives	2	Provide an explicit statement of the main objective(s) or question(s) the review addresses.	Yes
**METHODS**			
Eligibility criteria	3	Specify the inclusion and exclusion criteria for the review.	Yes
Information sources	4	Specify the information sources (e.g. databases, registers) used to identify studies and the date when each was last searched.	Yes
Risk of bias	5	Specify the methods used to assess risk of bias in the included studies.	Yes
Synthesis of results	6	Specify the methods used to present and synthesise results.	Yes
**RESULTS**			
Included studies	7	Give the total number of included studies and participants and summarise relevant characteristics of studies.	Yes
Synthesis of results	8	Present results for main outcomes, preferably indicating the number of included studies and participants for each. If meta-analysis was done, report the summary estimate and confidence/credible interval. If comparing groups, indicate the direction of the effect (i.e. which group is favoured).	Yes
**DISCUSSION**			
Limitations of evidence	9	Provide a brief summary of the limitations of the evidence included in the review (e.g. study risk of bias, inconsistency and imprecision).	Yes
Interpretation	10	Provide a general interpretation of the results and important implications.	Yes
**OTHER**			
Funding	11	Specify the primary source of funding for the review.	N/A

Section and Topic	Item #	Checklist item	Reported (Yes/No)
Registration	12	Provide the register name and registration number.	Yes

Table S4. Articles excluded in full-text review and reasons for exclusion

No.	Reference	Reasons for exclusion
1	Kittipongvises, S.; Phetrak, A.; Rattanapun, P.; Brundiers, K.; Buizer, J.L.; Melnick, R. AHP-GIS analysis for flood hazard assessment of the communities nearby the world heritage site on Ayutthaya Island, Thailand. *Int. J. Disaster Risk Reduct.* **2020**, *48*, 101612. https://doi.org/10.1016/j.ijdrr.2020.101612 [67].	Technical study without community assessment
2	Ehsan, S.; Begum, R.A.; Abdul Maulud, K.N.; Mia, M.S. Assessing household perception, autonomous adaptation and economic value of adaptation benefits: Evidence from West Coast of Peninsular Malaysia. *Adv. Clim. Chang. Res.* **2022**, *13*, 738–758. https://doi.org/10.1016/j.accre.2022.06.002 [68].	Outcome did not match
3	Lan, B.T.H.; Truong, D.D.; Dat, T.T.; Quang, B.H.; Hang, N.D.; Huan, L.H. Assessment of community livelihood vulnerability to climate change in Vietnam: A case study of ethnic groups in Northern Upland Region. *PLoS ONE* **2025**, 20, e0330482. https://doi.org/10.1371/journal.pone.0330482 [69].	Outcome did not match
4	Wongpanarak, N.; Langkulsen, U. Climate change and mental health in Northeast of Thailand. *Int. J. Environ. Health Res.* **2024**, *34*, 3860–3875. https://doi.org/10.1080/09603123.2024.2328741 [70].	Outcome did not match
5	Langkulsen, U.; Cheewinsiriwat, P.; Rwodzi, D.T.; Lambonmung, A.; Poompongthai, W.; Chamchan, C.; Boonmanunt, S.; Nakhapakorn, K.; Moses, C. Coastal erosion and flood coping mechanisms in Southern Thailand: A qualitative study. *Int. J. Environ. Res. Public Health* **2022**, *19*, 12326. https://doi.org/10.3390/ijerph191912326 [71].	Technical study without community assessment
6	Shariff, N.N.M.; Hamidi, Z.S. Community-based approach for a flood preparedness plan in Malaysia. *Jàmbá J. Disaster Risk Stud.* **2019**, *11*, a598. https://doi.org/10.4102/jamba.v11i1.598 [72].	Outcome did not match
7	Abid, S.K.; Sulaiman, N.; Al-Wathinani, A.M.; Goniewicz, K. Community-based flood mitigation in Malaysia: Enhancing public participation and policy effectiveness for sustainable resilience. *J. Glob. Health* **2024**, *14*, 04290. https://doi.org/10.7189/jogh.14.04290 [73].	Technical study without community assessment
8	Alias, N.A.; Mohd Idris, N.D.; Siwar, C.; Alam, M.M.; Yasar, M. Community preparation and vulnerability indices for floods in Pahang State of Malaysia. *Land* **2021**, *10*, 198. https://doi.org/10.3390/land10020198 [74].	Outcome did not match
9	Abid, S.K.; Sulaiman, N.; Samman, B.; Al-Wathinani, A.M.; Goniewicz, K. Cultural Dynamics in Disaster Management: Exploring Sociocultural Influences on Disaster Response in Malaysia. *Disaster Med. Public Health Prep.* **2025**, *19*, e69. https://doi.org/10.1017/dmp.2025.68 [75].	Not specific to community flood resilience
10	Isahak, A.; Reza, M.I.H.; Siwar, C.; Ismail, S.M.; Sulaiman, N.; Hanafi, Z.; Zainuddin, M.S.; Taha, M.R. Delineating risk zones and evaluation of shelter centres for flood disaster management along the Pahang River Basin, Malaysia. *Jàmbá J. Disaster Risk Stud.* **2018**, *10*, a501. https://doi.org/10.4102/jamba.v10i1.501 [76].	Technical study without community assessment

11	Allaire, M.C. Disaster loss and social media: Can online information increase flood resilience? *Water Resour. Res.* **2016**, *52*, 7408–7423. https://doi.org/10.1002/2016WR019243 [77].	Non-community level assessment
12	Heinkel, S.-B.; Thiebes, B.; Than, Z.M.; Aung, T.; Kyi, T.T.; Mar, W.L.; Oo, S.S.; Miller, C.; Willkomm, M.; Maung, W.; Myint, Z.N. Disaster preparedness and resilience at household level in Yangon, Myanmar. *Nat. Hazards* **2022**, *112*, 1273–1294. https://doi.org/10.1007/s11069-022-05226-w [78].	Non-community level assessment
13	Azmi, E.S.; How, V.; Rahman, H.A. Effect of health belief model on flood-risk educational approach among elementary school children in Malaysia. *Jàmbá J. Disaster Risk Stud.* **2021**, *13*, a1102. https://doi.org/10.4102/jamba.v13i1.1102 [79].	Technical study without community assessment
14	Wongboontham, T.; Nitivattananon, V.; Pal, I.; Nguyen, T.P.L. Enhancing urban flood resilience: The role and influence of socio-economics in the Chao Phraya River Basin, Thailand. *Int. J. Sustain. Dev. Plan.* **2023**, *18*, 3061–3076. https://doi.org/10.18280/ijsdp.181009 [80].	Technical study without community assessment
15	Sufian, A.; Chai, J.C.; Azman, H.; Abdul Aziz, N.A.; Fen, F.S.; Mohd Zamri, A.A. Evaluating flood resistance and resilience using property level flood risk adaptation (PLFRA) in Melaka. *NeuroQuantology* **2022**, *20*, 2409–2417. https://doi.org/ 10.14704/nq.2022.20.9.NQ44282 [81].	Non-community level assessment
16	Sun, F.; Ke, J.; Vivarakanon, P.; Aung, M.N.; Xia, Q.; Jiang, L. Flooding and cognitive health among middle-aged and older adults in Thailand: A case study of resilient city policy in Bangkok. *Ann. Glob. Health* **2025**, *91*, 49. https://doi.org/10.5334/aogh.4740 [82].	Outcome did not match
17	Boonyaratkalin, P.; Partiprajak, S.; Piaseu, N. Flood preparedness literacy and behaviors in community dwelling older adults. *Disaster Med. Public Health Prep.* **2021**, *15*, 452–457. https://doi.org/10.1017/dmp.2020.27 [83].	Non-community level assessment
18	Rachmawati, F.; Susilawati, C.; Teo, M.; Saja, A.M.; Devi, B.; Soemitro, R.A.; Wilkinson, S.; Goonetilleke, A. Flood risk perception, awareness, and preparedness behaviours among vulnerable population groups: implications for building community resilience. *Disasters* **2026**, *50*, e70029. https://doi.org/10.1111/disa.70029 [84].	Non-community level assessment
19	Srikuta, P.; Inmuong, U.; Inmuong, Y.; Bradshaw, P. Health vulnerability of households in flooded communities and their adaptation measures: case study in Northeastern Thailand. *Asia Pac. J. Public Health* **2015,** *27*, 743–755. https://doi.org/10.1177/1010539514568709 [85].	Outcome did not match
20	Nguyen, T.A.; Vu, D.A.; Vu, P.V.; Nguyen, T.N.; Pham, T.M.; Nguyen, H.T.; Trinh Le, H.; Nguyen, T.V.; Hoang, L.K.; Vu, T.D.; Nguyen, T.S. Human ecological effects of tropical storms in the coastal area of Ky Anh (Ha Tinh, Vietnam). *Environ. Dev. Sustain.* **2017**, *19*, 745–767. https://doi.org/10.1007/s10668-016-9761-3 [86].	Outcome did not match
21	Mutia, E.; Azmeri, A.; Yulianur, A.; Achmad, A.; Meilianda, E. Multifactor analysis of urban pluvial flooding using a comprehensive vulnerability index. *Jàmbá J. Disaster Risk Stud.* **2025**, *17*, a1835. https://doi.org/10.4102/jamba.v17i1.1835 [87].	Technical study without community assessment

22	Irvine, K.N.; Suwanarit, A.; Likitswat, F.; Srilertchaipanij, H.; Sahavacharin, A.; Wongwatcharapaiboon, J.; Boonkam, P.; Ingegno, M.; Janpathompong, S. Nature-based solutions to enhance urban flood resiliency: Case study of a Thailand Smart District. *Sustain. Water Resour. Manag.* **2023**, *9*, 43. https://doi.org/10.1007/s40899-023-00821-6 [88].	Technical study without community assessment
23	Langlois, B.K.; Ismanto, A.; Beaulac, L.; Berry, K.; Koch, M.; Griffin, T.; Coughlan de Perez, E.; Naumova, E.N. Recurrent flooding and household food access in Central Java, Indonesia. *Int. J. Environ. Res. Public Health* **2024**, *21*, 1370. https://doi.org/10.3390/ijerph21101370 [89].	Outcome did not match
24	Oza, H.H.; Salinger, A.P.; Taruc, R.; Tela, A.; Barker, S.F.; Leder, K.; Freeman, M.C.; Clasen, T.; Sinharoy, S.S. Resilience measurement for environmental shocks and stressors: scale development and psychometric assessment for coastal urban informal settlements in Fiji and Indonesia. *BMC Glob. Public Health* **2025**, *3*, 61. https://doi.org/10.1186/s44263-025-00177-3 [90].	Ineligible study design
25	Kurniawan, T.A.; Meidiana, C.; Goh, H.H.; Zhang, D.; Jiang, M.; Othman, M.H.; Anouzla, A.; Aziz, F.; Mahmoud, M.; Khan, M.I.; Ali, I. Social dimensions of climate-induced flooding in Jakarta (Indonesia): The role of non-point source pollution. *Water Environ. Res.* **2024**, *96*, e11129. https://doi.org/10.1002/wer.11129 [91].	Outcome did not match
26	Langkulsen, U.; Rwodzi, D.T.; Cheewinsiriwat, P.; Nakhapakorn, K.; Moses, C. Socio-economic resilience to floods in coastal areas of Thailand. *Int. J. Environ. Res. Public Health* **2022**, *19*, 7316. https://doi.org/10.3390/ijerph19127316 [92].	Ineligible study design
27	Saulnier, D.D.; Hean, H.; Thol, D.; Ir, P.; Hanson, C.; von Schreeb, J.; Mölsted Alvesson, H. Staying afloat: community perspectives on health system resilience in the management of pregnancy and childbirth care during floods in Cambodia. *BMJ Glob. Health* **2020**, *5*, e002272. https://doi.org/10.1136/bmjgh-2019-002272 [93].	Not specific to community flood resilience
28	Alim, S.; Haryanti, F.; Subandi, A.; Prabandari, Y.S. Training on modified model of programme for enhancement of emergency response flood preparedness based on the local wisdom of Jambi community. *Jàmbá J. Disaster Risk Stud.* **2019**, *11*, a757. https://doi.org/10.4102/jamba.v11i1.801 [94].	Ineligible study design
29	Setiawan, M.A.; Winastuti, R.; Hayat, D.M.; Christanto, N.; Rahmadana, A.D.; Ngurah, I.; Ndapareda, E.; Swastanto, G.A.; Amri, I. Tropical Cyclone Seroja's aftermath: flash flood inundation modeling and recovery efforts in rural coastal Lembata, East Nusa Tenggara Province, Indonesia. *Trop. Cyclone Res. Rev.* **2025**, advance online publication. https://doi.org/10.1016/j.tcrr.2025.11.010 [95].	Outcome did not match
30	Dwirahmadi, F.; Rutherford, S.; Phung, D.; Chu, C. Understanding the operational concept of a flood-resilient urban community in Jakarta, Indonesia, from the perspectives of disaster risk reduction, climate change adaptation, and development agencies. *Int. J. Environ. Res. Public Health* **2019**, *16*, 3993. https://doi.org/10.3390/ijerph16203993 [96].	Technical study without community assessment
31	Saulnier, D.D.; Thol, D.; Por, I.; Hanson, C.; von Schreeb, J.; Mölsted Alvesson, H. 'We have a plan for that': a qualitative study of health system resilience through the perspective of health workers managing antenatal and childbirth services during	Non-community level assessment

	floods in Cambodia. *BMJ Open* **2022**, *12*, e054145. https://doi.org/10.1136/bmjopen-2021-054145 [97].	
